# The Role of Macrophages in Various Types of Tumors and the Possibility of Their Use as Targets for Antitumor Therapy

**DOI:** 10.3390/cancers17030342

**Published:** 2025-01-21

**Authors:** Enar Jumaniyazova, Anastasiya Lokhonina, Dzhuliia Dzhalilova, Ekaterina Miroshnichenko, Anna Kosyreva, Timur Fatkhudinov

**Affiliations:** 1 Research Institute of Molecular and Cellular Medicine, Peoples’ Friendship University of Russia (RUDN University), 6 Miklukho-Maklaya Street, 117198 Moscow, Russia; 2 Avtsyn Research Institute of Human Morphology, FSBSI Petrovsky National Research Centre of Surgery, 3 Tsyurupy Street, 117418 Moscow, Russia; 3 National Medical Research Center for Obstetrics, Gynecology and Perinatology Named after Academician V.I. Kulakov, Ministry of Healthcare of Russian Federation, 4 Oparina Street, 117997 Moscow, Russia

**Keywords:** tumor-associated macrophages, tumor microenvironment, immunotherapy, cancer, lung cancer, breast cancer, head and neck squamous cell cancer, soft tissue sarcomas

## Abstract

In solid tumors, tumor-associated macrophages (TAMs) are one of the most numerous populations and play an important role in the processes of tumor cell invasion, metastasis, and angiogenesis. Therefore, TAMs are considered promising diagnostic and prognostic biomarkers of tumors, and many attempts have been made to influence these cells as part of antitumor therapy. There are several key principles of action on ТАМs: the inhibition of monocyte/macrophage transition; the destruction of macrophages; the reprogramming of macrophage phenotypes (polarization of M2 macrophages to M1); the stimulation of phagocytic activity of macrophages and CAR-M therapy. Despite the large number of studies in this area, to date, there are no adequate approaches using antitumor therapy based on alterations in TAM functioning that would show high efficacy when administered in a mono-regimen for the treatment of malignant neoplasms. Studies devoted to the evaluation of the efficacy of drugs acting on TAMs are characterized by a small sample and the large heterogeneity of patient groups; in addition, in such studies, chemotherapy or immunotherapy is used, which significantly complicates the evaluation of the effectiveness of the agent acting on TAMs. In this review, we attempted to systematize the evidence on attempts to influence TAMs in malignancies such as lung cancer, breast cancer, colorectal cancer, cervical cancer, prostate cancer, gastric cancer, head and neck squamous cell cancer, and soft tissue sarcomas.

## Simple Summary

Macrophages are the largest cell population among immune cells in the tumor microenvironment. They are characterized by plasticity and high sensitivity to environmental changes. In tumor tissue, a larger percentage of macrophages are characterized by a protumor phenotype, and it is this nuance that makes these cells a promising target for intervention to halt tumor progression. In this review, we have tried to describe the different subtypes of macrophages and their role in tumorigenesis, as well as to describe the methods of action on macrophages in some types of malignancies.

## 1. Introduction

Macrophages are the main cells of innate immunity. They originate from blood monocytes. One of the main functions of monocytes and macrophages is the regulation of inflammation. In order to effectively perform their functions, macrophages acquire a phenotype characteristic of a specific microenvironment during differentiation, i.e., they are able to adapt to changing conditions [1]. In solid tumors, tumor-associated macrophages (TAMs) are one of the most numerous populations [2] and play an important role in the processes of tumor cell invasion, metastasis, and angiogenesis [3]. Therefore, TAMs are considered promising diagnostic and prognostic biomarkers of tumors, and many attempts have been made to influence these cells as part of antitumor therapy.

## 2. Types of Tumor-Associated Macrophages

Macrophages are able to change their phenotype and functional spectrum depending on the microenvironment, thereby demonstrating heterogeneity of subpopulations. Polarization of macrophages plays an important role in the development of a number of diseases, including tumors. Activated macrophages are often classified as proinflammatory M1 macrophages or anti-inflammatory M2 macrophages.

The M1 phenotype is induced by Toll-like receptor ligands (e.g., LPS) or Th1 cytokines such as TNF-α, IFN-γ, and colony-stimulating factor 2 (CSF2). Classical M1 macrophages are characterized by surface expression of TLR2, TLR4, CD80, and CD86 [4,5]. M1 macrophages have a high antigen-presenting capacity [6]. They secrete reactive oxygen species (ROS) and proinflammatory cytokines such as interleukins IL-1, IL-6, IL-12, IL-18, IL-23, and TNF-α, which modulate Th1-mediated antigen-specific inflammatory responses [7,8,9]. It has also been demonstrated that M1 macrophages enhance inducible nitric oxide synthase (NOS2 or iNOS) expression, promoting NO production from L-arginine [10]. The infiltration of M1 macrophages is considered a favorable prognostic factor for the course of tumor processes [11,12]. In contrast, M2 macrophages, induced by factors such as IL-4, IL-13, IL-10, or glucocorticoids, produce anti-inflammatory cytokines, the key ones being TGF-β and IL-10 [13]. By producing these cytokines, M2 macrophages create an immunosuppressive environment, which is why M2 macrophages are most often classified as TAMs [14,15]. TGF-β and IL-10 produced by macrophages inhibit cytotoxic T lymphocytes, as well as Th1 and Th2 CD4+ T cells [16]. However, there are also experimental data suggesting that TAMs are both M1 and M2 macrophages [17]. M2 macrophages have angiogenic and proinvasive properties; they produce growth factors, chemokines, and MMPs that act on tumor cells or microenvironment cells, stimulating tumor growth, invasion, and metastasis [18]. In this case, the stimulation of tumor cell extravasation and the maintenance of their stable growth in secondary foci is of great importance. Each of these processes is regulated by different subpopulations of macrophages [19]. These data, together with experimental studies demonstrating the inhibition of tumor progression and metastasis by depleting the macrophage population, confirm that the interaction of tumor cells with immune component cells plays a significant role in the acquisition of an aggressive phenotype by the former.

**Figure 1:**
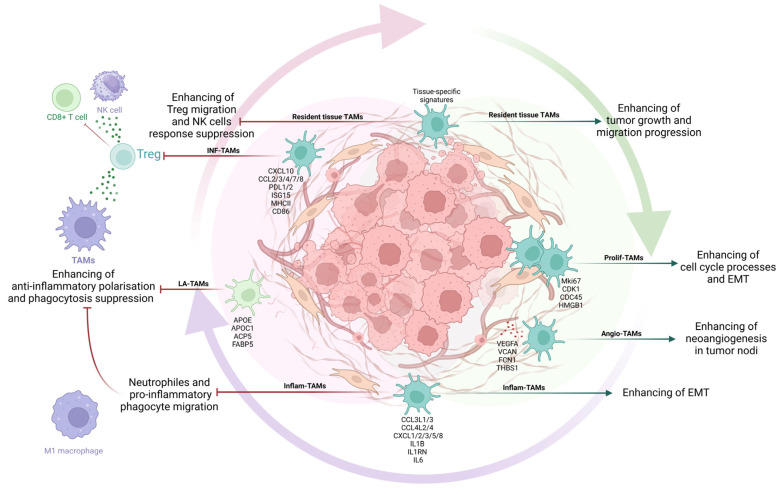
Types of tumor-associated macrophages.

The M1-M2 classification of macrophages has been criticized in recent years, since macrophages represent a dynamic population of cells in which the expression of genes can change depending on the microenvironment [8,20]. Based on studies using sequencing, a consensus model of the transcriptomic diversity of TAMs is currently being formed. According to the obtained data, based on signature genes, signaling pathways, and functions in the macrophage population, seven main subtypes can be distinguished: interferon-primed TAMs (IFN-TAMs), immune regulatory TAMs (Reg-TAMs), inflammatory cytokine-enriched TAMs (Inflam-TAMs), lipid-associated TAMs (LA-TAMs), proangiogenic TAMs (Angio-TAMs), RTM-like TAMs (RTM-TAMs), and proliferating TAMs (Prolif-TAMs) [21] (Figure 1).

IFN-TAMs are characterized by high IFN-regulated expression of genes such as *CXCL10* [22,23], programmed death-ligand 1 (PDL1) [24,25], and interferon-stimulated gene 15 (ISG15) [22], as well as M1-type markers such as CD86 and MHCII [24]. Among all TAM subpopulations, only IFN-TAMs have a proinflammatory phenotype similar to M1 macrophages in their morphofunctional properties. However, contrary to the widespread belief that M1 macrophages exert antitumor activity, IFN-TAMs have immunosuppressive properties, as they have been shown to suppress immune responses through tryptophan degradation and immunosuppressive regulatory T cell (Treg) recruitment [26].

Reg-TAMs are characterized by high arginase 1 (ARG1), Mannose receptor C-type 1 (MRC1), and CX3C motif chemokine receptor 1 (CX3CR1) expression and resemble alternatively activated macrophages [25,27,28]. These TAMs have been identified in breast, gastric, kidney, liver, bladder, fibrosarcoma, lung, ovarian, skin, glioma, lymphoma, melanoma [25,27,28], colon [29], and pancreatic [30] tumors. Notably, other TAM subsets can also exert immunosuppressive effects. For example, it has been shown that when IFN-TAMs are co-cultured with human peripheral blood mononuclear cells, the former can suppress the differentiation of T lymphocytes from peripheral blood mononuclear cells (PBMC) [31], which is consistent with predictions based on the NicheNet cell–cell interaction analysis [24]. Therefore, the authors [21] suggest that the selection of a subset of Reg-TAMs does not reflect their functional activity, but only a set of distinctive signature genes that they express during tumor development.

Inflam-TAMs produce proinflammatory cytokines and chemokines such as IL1B, CXCL1/2/3/8, CCL3, and CCL3L1 [22,29,32,33,34]. Chemokine (C-X-C motif) ligands—CXCL1, 2, and 3—bind to the chemokine receptor CXCR3 and activate the migration of granulocytes to the site of injury or infection [35]. CCL3L1 and CCL3 are ligands for CCR1, CCR3, and CCR5, which attract monocytes and lymphocytes during inflammation [36]. Therefore, one of the functions of Inflam-TAMs in the tumor microenvironment (TME) is to regulate immune cell migration. Factors produced by this subtype of macrophages act as chemoattractants for neutrophils and promote the formation of neutrophil extracellular traps (NETs). NETs are web-like structures composed of highly decondensed chromatin fibers with histone proteins, matrix metalloproteinase 9 (MMP9), neutrophil elastase, myeloperoxidase, and others. NETs contribute to inflammatory carcinogenesis, malignancy progression, cancer-related thrombosis, and resistance to radiation and chemotherapy.

LA-TAMs express genes-encoding lipoproteins, including Apolipoprotein C-I (APOC1), Apolipoprotein E (APOE), ACP5 (acid phosphatase 5), and fatty acid-binding protein (FABP5) [37,38,39]. Lipid uptake by tumor-derived macrophages has been shown to result in M2 polarization and an anti-inflammatory phenotype via activation of Arg1 expression [40]. Thus, LA-TAMs may suppress antitumor immune responses and possibly promote tumor progression.

Angio-TAMs are characterized by high expression of vascular endothelial growth factor A (VEGFA), secreted phosphoprotein 1 (SPP1), and other angiogenic factors such as Versican (VCAN), Ficolin-1 (FCN1), and Thrombospondin 1 (THBS1) [22,29,41]. In both humans and GL261 GBM mouse glioma models, it has been shown that angio-TAMs typically infiltrate tumor regions with the lowest oxygen content [42,43]. In vivo breast cancer models have shown that TAM-derived VEGFA can promote tumor metastasis by stimulating tumor cell intravasation and extravasation [44,45]. Thus, angio-TAMs promote tumor cell dissemination, and an increase in their number in the TME correlates with an unfavorable prognosis [22].

RTM-TAMs, or resident tissue macrophages, are characterized by high expression levels of signatures characteristic of the embryonic period of development and are detected in the peritumoral region rather than in tumor tissue [22,37,46]. Depending on the tumor location, the corresponding RTM-TAMs express gene signatures, characteristic of macrophages of this organ under normal conditions in the absence of a tumor process. For example, RTM-TAMs in hepatocellular carcinoma [24,47] and colorectal cancer liver metastases [29] express signatures characteristic of Kupffer cells, such as macrophage receptor with collagenous structure (MARCO), V-set and immunoglobulin domain-containing 4 (VSIG4), and FOLR2 (Folate receptor beta 2). Similarly, in lung cancer [23,48] or osteosarcoma lung metastases [32], RTM-TAMs are characterized by high levels of MARCO, scavenger receptor (SIGLEC1 (CD169)), and fatty acid-binding protein 4 (FABP4) expression, which is also characteristic of alveolar macrophages. In the Trp53fl/fl LSL-K-rasG12D (KP) mouse model of non-small-cell lung cancer [46] and glioblastoma [49], RTM-TAMs were shown to promote tumor invasiveness by inducing the epithelial–mesenchymal transition (EMT) of tumor cells and activating Treg migration into the tumor. Further studies are needed to identify differences between RTM-TAMs in different tumors.

Prolif-TAMs are characterized by the proliferation marker Ki-67 (MKI67) and cell cycle genes such as cyclin-dependent kinase 1 (CDK1) and cell division cycle 45 (CDC45) expression. Prolif-TAMs have been shown to express high levels of HMGB1, suggesting a proinflammatory phenotype [50]. Whether these cells represent a transient state that rapidly gives rise to other TAM subsets or whether they remain in the cell cycle as progenitors is unclear.

The above-described TAM subsets are not complete. In addition to these TAM subsets, there are others, specific to a certain tumor type. For example, [51] reported that scRNA-seq identified four new TAM subsets in various solid tumors based on core gene signatures, including FCN1+, SPP1+, C1Q+, and CCL18+ TAMs. FCN1+ TAMs can induce inflammation; SPP1+ TAMs are potentially involved in angiogenesis, metastasis, and tumor stem cell activation, while C1Q+ TAMs are involved in immune regulation and suppression. CCL18+ cells are terminal immunosuppressive macrophages which not only have a stronger immunosuppressive function but also enhance tumor metastasis. SPP1+ and C1Q+ TAM subsets can be further divided into distinct populations with distinct functions. However, there are some discrepancies between the cell types and subpopulations identified by scRNA-seq and their actual function, so further studies are needed.

Until a consensus model of macrophage transcriptome diversity has entered into popular use, in this review, we rely on the generally accepted classification of M1-M2 macrophages and describe methods for targeting TAMs in different types of malignancies.

## 3. Using Macrophages as Targets for the Treatment of Different Types of Cancer

It is now well known that a dynamic interaction is created between tumor cells and TME cells during tumor progression, which influences the clinical course of the disease, including the response to antitumor drugs, the rate of metastasis, the frequency of relapses, and ultimately, the overall survival of patients [52].

The fact that tumor cells remain undetected by the immune system has served as the basis for numerous studies aimed at investigating the subpopulations of cells infiltrating tumor tissue. The results obtained demonstrate the predominance of suppressor cell populations in the TME, and in particular, macrophages [53]. As mentioned above, a distinctive feature of macrophages is their plasticity, which allows them to respond to environmental signals and, depending on this, acquire various forms of polarization [54].

The protumor properties of TAMs make them promising therapeutic targets for the treatment of solid tumors [55]. First of all, we are talking about macrophages of the anti-inflammatory M2 phenotype, since they play one of the key roles in the processes of the EMT, invasion, and migration of cancer cells, and angiogenesis with subsequent tumor cell dissemination [56,57].

There are several key principles of action on ТАМs: the inhibition of monocyte/macrophage transition [58]; the destruction or depletion of macrophages [59]; the reprogramming of macrophage phenotypes (polarization of M2 macrophages to M1) [60]; and the stimulation of phagocytic activity of macrophages. Therapeutic approaches of antitumor treatment based on the above-mentioned principles of action on TAMs are at the stage of clinical studies assessing their efficacy and safety [61,62,63]. In addition to these principles of treatment of TAMs, CAR-M therapy is gaining popularity. The basic principle is that monocytes are isolated from the patient’s blood and then transduced with the desired antigen-specific chimeric receptor, for example anti-HER2, using proprietary viral or nonviral methods. Finally, the patient undergoes reinfusion of CAR-M [64]. The effects of HER2-directed CAR-M are being evaluated in clinical trials: a phase I trial (NCT04660929) in tumors overexpressing HER2; and a phase I trial (NCT06224738) registered in 2024 to evaluate the efficacy of HER2-CAR-M therapy for HER2-positive disseminated gastric cancer with metastases to the peritoneum [65].

As mentioned above, TAMs originate from blood monocytes, and this transition often occurs through activation of the chemokine receptor CCL-2-CC (CCR)-2 [66,67]. Thus, tumor and stromal cells of the TME synthesize CCL-2, which acts as a chemoattractant for monocytes expressing CCR-2 on their surface. Therefore, by influencing this signaling pathway, it is possible to influence the number of TAMs. Clinical trials aimed at assessing the antitumor efficacy of some CCR-2 inhibitors (PF-04136309, MLN1202, CCX872-B) and the CCL-2 blocker Carlumab (CNTO 888) are ongoing [62,68,69]. Interestingly, the addition of such agents to established polychemotherapy schemes has a positive impact on treatment outcomes. An increase in therapeutic efficacy was demonstrated when using a combination of FOLFIRINOX chemotherapy and the CCR-2 inhibitor PF-04136309 in patients with pancreatic cancer. According to instrumental diagnostic methods, in the combination treatment group, 16 of 33 patients showed an objective tumor response (49%), and 32 of these patients achieved local tumor control (97%) [70].

Another axis considered for treatment involving the recruitment of monocytes and their subsequent conversion to TAMs is the CXCL12/CXCR4 axis [45,71]. In preclinical models of breast cancer, prostate cancer, and ovarian cancer, blocking CXCR-4 has been shown to reduce overall tumor burden and metastatic activity [72].

Colony-stimulating factor-1 (CSF-1) plays an important role in the regulation of macrophage migration and survival. Increased expression of CSF-1 and CSF-1R is associated with a poor prognosis of malignant neoplasms [73]. Therefore, CSF-1R inhibitors are considered promising antitumor drugs and are currently undergoing clinical trials [74]. In preclinical studies on some tumor models, as well as in clinical trials, the combined use of CSF1/CSF1R inhibitors with various antitumor treatments—chemotherapy, immunotherapy, and radiotherapy [75,76,77,78]—did not show a significant therapeutic effect. For example, in a randomized phase II study in 50 patients with advanced triple-negative breast cancer, combination therapy with monoclonal antibodies against CSF1—Lacnotuzumab (MCS110) with Gemcitabine and Carboplatin—showed comparable antitumor efficacy to chemotherapy with Gemcitabine and Carboplatin alone [78]. In contrast, another randomized phase II trial of the combination of the anti-CSF1R monoclonal antibody Cabiralizumab with anti-PD-1 (Nivolumab) in patients with advanced pancreatic cancer who had received chemotherapy and no immunotherapy showed an increase in the duration of efficacy (ClinicalTrials.gov identifier: NCT02526017) [79]. To date, only one CSF1R inhibitor, Pexidartinib, has been approved by the FDA (Food and Drug Administration), but only for the treatment of tenosynovial giant cell tumors [80]. A study [81] in a mouse model of pancreatic cancer demonstrated the efficacy of combination therapy including the GVAX vaccine and anti-PD-1 and anti-CSF-1R antibodies. Combination therapy was associated with increased survival, the relative numbers of CD4+ and CD8+ T cells that co-expressed PD-1 and CD137, and the number of PD-1 + OX40+ CD4+ T cells in tumors.

An interesting fact is that some chemotherapeutic agents (which are actively prescribed to cancer patients) are able to change the polarization of TAMs and improve the tumor response to treatment. This ability is characteristic of Gemcitabine in pancreatic cancer [82], 5-Fluorouracil in colorectal cancer [83], and platinum-based chemotherapy in high-grade ovarian cancer [84]. Even more surprising is the discovery of the ability of Metformin (a hypoglycemic drug) to reduce the number of M2 macrophages in the TME and thereby suppress angiogenesis. Melatonin increases the number of M1 macrophages in the TME [85].

Rodriguez-Garcia et al. showed that the cytotoxic activity of CAR T cells leads to a decrease in the number of TAMs in tumors [86]. In tumor models in mice, CAR T cells targeting FRβ (expressed by M2 macrophages) led to selective elimination of FRβ+ macrophages, activated the migration of proinflammatory monocytes and tumor-specific CD8+ T cells, and ultimately slowed tumor growth [86].

In the following, we focus on the description of macrophage-targeting therapy in some types of malignancies (Table 1).

## 4. TAMs and Lung Cancer

Lung cancer (LC) is the second most common and the most lethal cancer in the world. LC is divided into two main types, including small-cell lung cancer (SCLC) and non-small-cell lung cancer (NSCLC), the latter type accounting for about 80–85% of all LC cases [116]. The most well-known risk factors for LC are active and passive smoking, alcohol consumption, exposure to asbestos, radon, arsenic, ionizing radiation, or polycyclic aromatic hydrocarbons, and family history [117,118]. An FDA-approved drug for the treatment of metastatic NSCLC is Lurbinectedine, a cellular transcription inhibitor [119]. Lurbinectedine induces selective apoptosis of TAMs and reduces the migration of monocytes and specific inflammatory mediators (CCL2, IL6, CXCL8). Also, Lurbinectedine reduces angiogenesis and immunosuppression and increases the infiltration of T cells expressing IFNγ and PD1, thereby improving the response to immunotherapy [87].

NSCLC is more common, with more than 55% of all patients diagnosed at late stages of the disease [120]. For example, Nivolumab, a human monoclonal antibody that blocks the interaction between PD-1 and its ligands (PD-L1 and PD-L2), is approved for the treatment of NSCLC. PD-L1, a PD-1 ligand, is expressed on tumor cells, APCs, T lymphocytes, and macrophages. PD-1 expression on TAMs was negatively correlated with tumor cell phagocytosis, and the blockade of PD-1/PD-L1 in vivo enhanced phagocytosis of macrophages and reduced tumor growth [99]. Atezolizumab, Pembrolizumab, and Durvalumab are also approved for the treatment of NSCLC.

The CSF1/CSF1R and CCL2/CCR2 axes, as well as CCL5 and VEGF, are classical regulatory factors that influence monocyte recruitment to tumors and shape their function in the TME [121]. Targeting these factors results in decreased macrophage recruitment. The clinical trial NCT02323191 evaluated the efficacy and safety of Emactuzumab (CSFR-1 inhibitor) in combination with Atezolizumab in patients with metastatic NSCLC. The results showed that the rate of confirmed objective response was 12.5%, and the safety profile of the drug combination was favorable, indicating great promise for the combination of CSFR-1 inhibitors with PD-L1 inhibitors.

At the same time, the mechanisms of resistance to immunotherapy remain a major problem—the absence of neoantigens or abnormal antigen presentation, low tumor load, low PD-L1 expression, impaired T cell infiltration or T cell depletion, the presence of immunosuppressive cells or factors, and in particular the predominance of TAMs in the TME [122]. This is why the search for new potential targets for cancer therapy continues. For example, there is evidence that the suppression of CD47/SIRPα signaling pathway components leads to the activation of tumor cell phagocytosis by macrophages [123], and high CD47 expression correlates with low PFS (progression-free survival) and OS (overall survival) rates [124]. Scientists’ expectations are focused on a novel agent, Eganelisib (IPI-549), which is a PI3K-γ inhibitor. Eganelisib reprograms M2 macrophages into M1 macrophages, thereby reducing the immunosuppressive potential of the TME and promoting the migration and proliferation of cytotoxic T cells. The clinical trial NCT02637531 which aims to evaluate the efficacy and safety of Eganelisib (monotherapy or in combination with Nivolumab) in patients with NSCLC is in the enrolment phase.

Also of interest is the blockade of IL10, an anti-inflammatory cytokine whose high expression in TAMs in NSCLC correlates with tumor stage, size, lymph node metastasis, lymphovascular invasion, and differentiation grade [125,126,127]. To remodel the immunosuppressive TAM-predominated environment, CRISPR knockout of the anti-inflammatory cytokine IL37 was used. It resulted in restoration of the cytolytic activity and antitumor capacity of NK cells and T cells and decreased Treg cell activity. In a mouse Lewis carcinoma model, the inhibition of USP7 was shown to mediate TAM reprogramming to M1 macrophages via activation of the p38 MAPK pathway [101].

## 5. TAMs and Breast Cancer

Breast cancer (BC) is the most common malignant tumor in women. The incidence of BC has increased worldwide in recent decades, especially among young women, and this is what is alarming [128]. Among the etiologic factors of BC are a previous history of the disease in a woman or a history of ВС in first-line relatives, obesity and a sedentary lifestyle, tall stature, early menarche and late menopause, a lack of childbirth and lactation, smoking, and alcohol consumption; the use of hormone replacement therapy is also considered a factor by some. The above-mentioned increase in the incidence of BC (by 0.5% per year) is primarily due to factors such as obesity and decreasing fertility among women [129].

According to a meta-analysis [130], a high number of TAMs were found in primary BC of all stages in 2000 patients, and their infiltration density correlated with an increased metastatic potential and the risk of tumor progression [131]. TAMs in BC are predominantly represented by a subpopulation with the M2 phenotype, characterized by protumor activity [132]. A high number of CD163+ TAMs in primary BC correlates with unfavorable clinicopathological characteristics [133,134].

A study [135] in a transplantable p53-null mouse model demonstrated that macrophages migrate primarily to areas of ductal hyperplasia with high tumor-forming potential, where they differentiate and polarize toward the protumor M2 phenotype.

TAMs play a key role in the formation of resistance to antitumor treatment in BC. The secretion of IL-10 by TAMs, which is responsible for regulating the expression of BCL-2 and STAT3, causes activation of the IL-10-STAT3-BCL2 pathway in BC, which increases resistance to antitumor drugs [136]. TAMs can increase the level of FABP5 and PPAR (peroxisome proliferator-activated receptors) in BC cells, activating the CaMKII (Ca^2+^/calmodulin-dependent protein kinase II) signaling pathway and leading to resistance to Doxorubicin (which is a commonly prescribed drug for BC) [137]. TAMs increase the secretion of chemokine CXCL5, which not only recruits monocytes but also activates the PI3K/AKT/mTOR pathway in tumor cells, mediating therapeutic resistance and tumor cell survival [92]. Activation of the PI3K/AKT/mTOR signaling cascade in tumor cells leads to a feedback increase in sodium–glucose co-transporter (SGLT) 1 and activates glycolysis, thereby promoting Tamoxifen resistance and accelerating tumor growth both in vitro and in vivo [138]. M2 TAMs can reduce the efficacy of Paclitaxel via activation of the IL-10/STAT3/Bcl-2 signaling pathway and can cause BC cells to become resistant to the drug [139]. In addition to chemoresistance, TAMs cause resistance to immunotherapy by suppressing T cell function [140].

Activation of the CSF-1/CSF-1R pathway or higher expression of CSF-1 or CSF-1R results in a poor prognosis of BC in postmenopausal women [141]. Blocking the CSF1/CSF1R pathway suppresses macrophage migration into tumor tissue. Administration of CSF-1 antisense oligodeoxyribonucleotide and siRNA directed against CSF-1 mRNA or host (mouse) CSF-1 receptors to mice bearing human breast cancer xenografts suppressed tumor growth by 40–50% and increased mouse survival. In addition, selective reduction in MMP 2, MMP 12, and VEGF-A expression was observed, which indicated the inhibition of tumor angiogenesis [142]. CSF1R inhibitors have been shown to enhance the efficacy of chemotherapy and radiotherapy in experimental models of BC [143]. However, blocking CSF1R with monoclonal antibodies or small-molecule inhibitors not only suppresses M2 macrophages but also affects the activity of M1 macrophages. Therefore, further studies aimed at finding approaches to selectively deplete only M2 macrophages are required. That is probably why the inhibition of CSF1R has not led to the desired results in several clinical trials. In advanced solid tumors, anti-CSF1R Emactuzumab failed to produce objective clinical responses either as monotherapy or in combination with Paclitaxel or a CD40 agonist (Selicrelumab) [144,145]. Phase III clinical trials have shown that in the common triple-negative subtype of ВС, the CSF-1R inhibitors PLX3397, LY3022855, and Cabiralizumab enhance CD8+ T cell activity [59]. However, despite good tolerability and initially favorable effects, the main limitation for the introduction of these agents into the clinic is the rapid development of resistance to them [146]. Several clinical trials have evaluated the efficacy and safety of Pexidartinib and PLX3397 (targeting the CSF1/CSF-1R axis) in combination with Eribulin (NCT01596751) and Paclitaxel (NCT01525602) in patients with metastatic BC, but the results are difficult to assess due to the small patient sample.

The CXC chemokine subfamily is an important regulator of macrophage recruitment to tumors. CXCL12, a member of the CXC chemokine subfamily, is released by stromal cells and fibroblasts in BC [147]. Treatment of BC with antibodies against CXCL12 resulted in decreased levels of CD163 and VEGFA mRNA expression in TAMs and decreased numbers of M2-type macrophages and suppressed angiogenesis [91].

The study by Ball et al. investigated the effect of the synthetic oleanane triterpenoid CDDO-methyl ester on TAM functions in vivo. PyMT+/− female mice that were treated with CDDO-Me administration showed a decrease in TAM concentration compared to control group mice [90].

The use of a DNA vaccine against Asparaginyl Endopeptidase Legumain (which is overexpressed in TAMs) demonstrated a reduction in the number of TAMs in the TME of BC in syngeneic BALB/c mice and led to a decreased rate tumor growth, angiogenesis, and metastasis [89].

MicroRNAs (miRNAs) represent a new class of therapeutic agents aimed at regulating multiple signaling pathways within the TME [148]. Wang et al. found that miR-100 overexpression promoted the phenotype of TAMs through regulation of the mTOR pathway. Further, the team of scientists, by inhibiting miR-100 in TAMs in a mouse model of BC, managed to weaken the protumor potential of TAMs, which was accompanied by effects such as the suppression of tumor metastasis and increasing its chemosensitivity [149].

Chemotherapeutic agents such as Doxorubicin and Docetaxel, which are among the most commonly prescribed drugs for the treatment of BC, have the ability to suppress the activity of TAMs. Another drug, Paclitaxel, disrupts the polarization of M2 macrophages and reprograms TAMs to M1 macrophages via the TLR4/NF-κB signaling cascade [103].

Bisphosphonates prescribed for bone metastases in patients with BC also have an effect on TAMs. Bisphosphonates induce apoptosis of TAMs, suppress the release of proangiogenic factors, and inhibit the proliferation and migration of macrophages [150]. Administration of liposome-conjugated zoledronic acid resulted in the depletion of TAMs and the suppression of angiogenesis and primary tumor growth in triple-negative BC [88].

In a mouse model of four T1 BCs, Anemoside A3, an active compound of the perennial herb Pulsatilla, was shown to activate macrophage polarization toward M1 macrophages via the TLR4/NF-κB/MAPK pathway, suppressing BC progression [102].

Bindarit is an original compound with anti-inflammatory activity due to selective inhibition of monocyte chemotactic proteins CCL2, CCL7, and CCL8. In syngeneic Balb/c mice injected under the mammary gland with murine BC cells (4T1-Luc cells), Bindarit treatment significantly decreased the infiltration of TAMs and MDSCs [151].

Currently, phase 1 of the clinical trial of the anti-HER2 CAR macrophage CT-0508 in subjects with HER2-overexpressing solid tumors, including BC, is underway (NCT04660929).

## 6. TAMs and Colorectal Cancer

Colorectal cancer (CRC) remains the third most common malignancy, but is second only to lung cancer in mortality [152]. Most CRC cases are sporadic, but approximately five percent of CRC cases are due to a hereditary predisposition, mainly Lynch syndrome (hereditary nonpolyposis CRC) and familial adenomatous polyposis [153]. Non-hereditary modifiable risk factors include obesity, a sedentary lifestyle, eating large amounts of red processed meat, inadequate fiber intake, smoking, and alcohol abuse [154].

The role of TAMs in CRC is controversial. Some researchers argue that higher macrophage infiltration correlates with advanced disease stages [155] and a worse prognosis [156], while others report that TAMs are associated with prolonged patient survival rates [157] and reduced liver metastases [158]. Exosomes secreted by M2 macrophages promote metastasis by transferring specific miRNAs into CRC cells [159]. A study including 76 patients with CRC found a correlation between macrophage abundance and microvessel density in the tumor [160], which is in favor of the active participation of TAMs in angiogenesis. Tacconi et al. found that TAMs express VEGFR3 and promote CRC progression via the VEGFC/VEGFR3 axis [161].

Cetuximab (an IgG1 monoclonal antibody that binds to the extracellular domain of the EGFR and is used in disseminated CRC) repolarized TAMs from M2 to M1 phenotypes, mainly by suppressing IL-6 expression through NFκB and STAT3 pathways [104].

Six1 (sine oculis homeobox 1), a protein synthesized by cancer cells and inducing the infiltration of tumor tissue macrophages, has become the focus of interest of researchers searching for the “control button” of TAMs in CRC. Suppression of the *Six1* gene limited the proliferation and motility of CRC cells by suppressing the expression of macrophage-recruiting factors, such as CSF-1, CCL-2/5, and VEGF [162,163].

Given the important role of the CSF1/CSF1R axis in TAM recruitment and survival, CSF1R inhibition in combination with PD-1 inhibition has been attempted in patients with CRC. AMG 820 is a human IgG2 monoclonal antibody directed against CSF1R. However, antitumor activity was insufficient to further evaluate this combination in patients [164].

Wang et al. demonstrated the effect of methionine enkephalin on CRC, and its mechanisms of action were examined in vivo [165]. The intraperitoneal administration of methionine enkephalin effectively inhibited MC38 subcutaneous CRC growth in mice. This agent increased the infiltration of M1-type macrophages, CD8 T cells, and CD4 T cells, while decreasing the number of M2-type macrophages [165].

Maraviroc is a CCR5 receptor antagonist licensed for the treatment of HIV infection. In a phase I study, monotherapeutic inhibition of MARACON CCR5 (NCT01736813) resulted in macrophage repolarization toward an M1-like phenotype, thereby promoting an immune environment that inhibits CRC [105]. This drug therapy resulted in disease stabilization, and responses were observed in selected patients with a favorable toxicity pattern. In addition, a better response to subsequent chemotherapy was observed. However, in the PICCASSO clinical trial (NCT03274804), the efficacy and safety of Pembrolizumab and Maraviroc in refractory CRC were not confirmed. Clinical efficacy in patients with CRC was limited, but durable disease stabilization was observed in selected patients [166].

## 7. TAMs and Prostate Cancer

Prostate cancer (PC) is the second most common cancer in men worldwide. Surgery and radiation are the standard primary treatment for patients with early localized prostate malignancies, followed by androgen deprivation therapy (ADT), like surgical or chemical castration if the disease recurs [167]. Hormonal ADT is used for metastatic hormone-sensitive PC [168,169], but almost all PC patients eventually progress to incurable metastatic castration-resistant PC (mCRPC) [170,171].

Despite progress in the treatment of PC, the efficacy of current immunotherapy strategies, in particular checkpoint inhibition, in metastatic PC patients is limited [170]. Of great interest is therapy targeting TAMs, which are a common type of immunosuppressive cell in the TME. It has been shown that the infiltration of CD163+ M2 macrophages into PC tissue is associated with an unfavorable prognosis [172], which is due to the formation of an immunosuppressive environment. Hormonal therapy for PC is represented by androgen blockades. Enzalutamide, an androgen receptor (AR) inhibitor, is FDA-approved for the treatment of PC, but it is known to increase the expression level of HMGB1 in PC, which can attract and activate M2 TAMs. HMGB1-activated M2 TAMs additionally promote neuroendocrine differentiation (NED) of PC cells through IL-6 secretion [173], and NED usually indicates a poor prognosis with limited treatment options for patients with PC [173].

A study [174] showed that the number of M2 TAMs was significantly increased in ADT-treated mice, which contributed to the aggravation of immunosuppression. In addition, AR inhibition promotes PC cell migration/invasion via CCL2-dependent activation of STAT3 and EMT pathways [175]. A CCL2-CCR2 blockade can reduce TAM infiltration, but anti-CCL2 therapy can also promote metastasis [176]. High levels of growth factors and cytokines, such as TGF-β, IL10, and Arg1, as well as chemokines, CXCL2, CXCL8, and CXCL12 [177], attract other immunosuppressive cells, in particular Tregs [172], thereby promoting tolerance and tumor evasion by suppressing proinflammatory CD4+ type 1 (Th1) helper T cells and CD8+ cytotoxic T cells [178]. A clinical trial (NCT00992186) designed to determine the efficacy and safety of Carlumab (CCL2 antibody) in patients with metastatic refractory PC demonstrated that Carlumab was well tolerated but failed to show single-agent antitumor activity.

It has been previously shown that activation of the CSF-1/CSF-1R signaling pathway and overexpression of CSF-1R promote PC progression and increase the expression of the SPP1 transcript encoding osteopontin, a key factor in cancer development and metastasis [179]. Sun et al. reported that the use of sialic acid-targeted nanoparticles delivering CSF-1R siRNA resulted in the reprogramming of macrophages to the M1 phenotype in human and mouse PC models [108].

## 8. TAMs and Cervical Cancer

Cervical cancer (CC) is one of the most common female malignant tumors and a prevalent cause of malignant tumor-related death in women worldwide [180]. It is caused almost exclusively by oncogenic strains of human papillomavirus (HPV). Screening and HPV vaccination are the most effective methods to reduce CC mortality in women.

CC is characterized as an immune infiltrated but immunosuppressive cancer type, primarily due to the modulation of the TME by HPV [181,182]. One of the most abundant immune cell populations in the CC TME are macrophages. CC progression correlates with the infiltration of CD68+ and CD163+ M2 TAMs. CD163+ M2 TAM infiltration is associated with advanced CC and lymph node metastasis. CD204+ M2 macrophages may predict a poor prognosis in patients with cervical adenocarcinoma [183]. M2-type TAMs may upregulate the expression of PD-L1 on CC cells through the PI3K/AKT pathway, thus affecting the progression of tumors.

In CC, TAM tumor infiltration produces IL-10, which promotes metastasis [109,184]. Another study showed that under hypoxic conditions, increased production of Zinc finger E-box-binding homeobox 1 (ZEB1)—which downregulates E-cadherin and induces EMT in CC cells—promotes metastasis and disease progression by activating TAM migration into the tumor and forming a prometastatic environment in it [185].

In another study [186], Gene-Set Enrichment Analysis in CC cells revealed that the altered expression of genes was enriched in the NF-κB pathway and immune–inflammatory response pathways. Moreover, overexpression of tumor suppressor cyclin-dependent kinase 12 (CDK12) enhances TAM infiltration by immunohistochemistry techniques and gene expression profiling. So, CDK12 may contribute to the regulation of the TME in CC cells.

One of the mechanisms of antitumor immunity may be ferroptosis—a novel nonapoptotic form of regulated cell death, which involves iron-dependent lipid peroxide accumulation and causes lethal damage to cells [187], especially in cases where resistance to chemotherapy-induced apoptosis develops [188]. Ref. [180] found that TAMs can inhibit ferroptosis in CC cells through the secretion of exosomes containing miRNA-660-5p. miRNA-660-5p reduces the expression of the tumor suppressor gene ALOX15, a member of the lipoxygenases family, the lipid-peroxidizing enzymes [189].

Thus, a negative prognosis correlates with an increase in tumor infiltration of TAMs, which regulates proliferation and the death of tumor cells, and also participates in the regulation of EMT. Therefore, therapy aimed at reducing the number of anti-inflammatory TAMs and/or polarizing them to the M1 proinflammatory phenotype in CC may be promising. Thus, it has been shown that radiotherapy of cervical cancer induces an increase in the number of TAMs and a change in their subtype from the M2-like phenotype to the M1-like phenotype [109].

One of the novel therapeutic targets for treating CC may be Oct4—a homeodomain transcription factor of the POU family—which maintains embryonic stem cells’ state [190]. Oct4 activated by HPV facilitates CC cell growth via inhibiting p53 expression [191]. Oct4 has been shown to induce macrophage polarization in M2 macrophages, thereby maintaining the immunosuppressive potential of the CC TME [192]. Ref. [193] showed that Oct4 transcriptionally activates IL-17A to regulate the p38 signaling pathway and promotes M2 macrophage polarization, thereby promoting CC metastasis.

Poly methyl methacrylate (PMMA) is a synthetic polymer approved by the FDA for certain human clinical applications such as bone cement. PMMA 4 particles stimulated the highest level of TNF-α production by macrophages in vitro and gave the best result of antitumor protection in vivo.

## 9. TAMs and Gastric Cancer

Gastric cancer (GC) remains the fifth most common malignant cancer and the fourth leading cause of cancer-related deaths [152]. Two major anatomical subtypes of GC are distinguished, non-cardiac and cardiac, with different time trends and leading risk factors, such as obesity and reflux for cardiac GC, and helicobacter pylori infection for non-cardiac GC [194]. Due to the absence of specific symptoms at early stages, more than 80% of hospitalized patients are diagnosed with GC at locally advanced or metastatic stages [195]. Among the cells of the GC TME, macrophages are considered to play an important role in tumor progression and resistance to antitumor drugs. Sun et al. demonstrated that high expression of the TAMs CD68 or M2 CD206 usually predicted low overall survival, while high expression of M1 macrophages (CD86) is associated with a favorable prognosis in HER2-positive patients with GC [111]. Patients with GC and duodenal cancer with high expression of CD163 had low overall survival compared to patients with low expression of this marker [196]. Patients with high infiltration of CD204 macrophages in the TME of GC had a low 5-year survival rate [197]. At the same time, studies have shown that macrophages of the M1 phenotype suppress tumor cell viability and enhance the sensitivity of GC cells to chemotherapy [198]. Unfortunately, among cells of the TME in GC, it is the M2-phenotype macrophages that prevail, creating all the necessary conditions for rapid tumor progression. It has also been revealed that CD163+ TAMs in GC are associated with the increased density of microvessels in tumor tissue, indicating that M2-type TAMs can promote angiogenesis in GC [196,199,200,201].

M2 macrophages activate PI3K/AKT/mTOR and JAK1/STAT3 signaling cascades in cancer cells, and this leads to the progression of GC and the development of resistance to 5-fluorouracil [202,203]. Also, TAMs reduce the sensitivity of GC cells to Oxaliplatin and Doxorubicin by producing EVs containing circular RNA 0008253 37 and miR-223 [204].

There are not many studies aimed at researching or developing methods for the treatment of GC that would consider TAMs as a target. Since one of the possible options for antitumor treatment of GC is the administration of antiangiogenic therapy, attempts were made in a previous study [110] to prolong its effectiveness. The researchers created a bispecific fusion protein (mAb04-MICA). It consisted of an antibody targeting VEGFR2 fused to MICA α 1–α 2 ectodomain. mAb04-MICA inhibited GC cell proliferation through specific binding to VEGFR2 and induced the repolarization of TAMs from type M2 to type M1 both in vitro and in vivo [110].

Increased CCL2 expression may enhance TAM transition from the M1 to M2 phenotype and contribute to the development of Trastuzumab resistance in HER2-positive patients with GC. CCX140-B (MedChemExpress, USA) treatment, as a specific CCR2 inhibitor, efficiently blocked CCL2-CCR2 signaling, confirming the efficacy of CCL2 for TAMs [111].

When GC cells are co-cultured with M2 macrophages, the latter secrete MMP9 via increased COX-2 expression, promoting angiogenesis and GC invasion. Combination treatment with EGFR and COX-2 inhibitors inhibits GC tumorigenesis in transgenic mice [205].

Methionine enkephalin, which has demonstrated efficacy against CRC, in GC also promotes the transition of TAMs from type M2 to type M1 and induces cells apoptosis though blocking the OGFr/PI3K/AKT/mTOR signaling pathway [112].

Zhuang et al. demonstrated that sophoridine, an alkaloid extracted from the seeds of *Sophora alopecuroides* L., upregulated IL-12α and TNF-α, while it downregulated IL-10 and CD206 via the TLR4/IRF3 signaling pathway in the TME of GC, suggesting that sophoridine promoted TAMs in GC to polarize toward the M1 type and suppressed M2-type polarization [113].

Clever-1 is a multifunctional scavenger and adhesion receptor expressed by monocytes, M2 macrophage subsets, and some other cells [206]. High Clever-1 expression is associated with a poor prognosis, T-lymphocyte exclusion, impaired antigen presentation, and resistance to immune checkpoint inhibitors in some types of cancer. Bexmarilimab (FP-1305) is a humanized IgG4 monoclonal antibody specific to Clever-1 which has been shown to reduce the M2/M1 ratio in tumor tissue from patients with GC [94].

## 10. TAMs and Head and Neck Tumors

Among head and neck tumors, head and neck squamous cell cancer (HNSCC) accounts for 90%. HNSCC is the seventh most common cancer worldwide, but the incidence and mortality rates vary widely in different regions of the world. The main risk factors for HNSCC are tobacco smoking, alcohol consumption, areca nut consumption, HPV infection (especially for oropharyngeal cancer), and Epstein–Barr virus infection [207].

The TME of HNSCC is characterized by an abundance of TAMs: they account for up to 30% of all tumor-infiltrating cells. It is believed that tumors with such an “immune portrait” are characterized by a short period of relapse-free survival and low overall survival of patients [208,209], which is the characteristic nature of this type of cancer. Wu et al. synthesized a STING (stimulator of interferon genes) agonist linked via a cleavable linker to antibodies targeting the EGFR. Having tested the resulting conjugate in mouse models, the authors discovered an immunomodulatory effect consisting of the repolarization of TAMs from the M2 to M1 phenotype [114].

An unusual effect on TAMs was found with lipid-lowering statin drugs. Statins inhibited the proliferation of HNSCC cells, simultaneously enhancing the antitumor cytotoxicity of T lymphocytes and promoting the repolarization of macrophages from the M2 to M1 phenotype [210].

Signal transducer and activator of transcription 3 (STAT3) is overexpressed in various cancers and acts as a critical signaling hub in tumor cells and cellular components of the TME, especially tumor-infiltrating immune cells [211], controlling cell proliferation, migration, and apoptosis. Moreira et al. found that targeting STAT3 in TAMs could enhance the therapeutic effects of radiotherapy in HNSCC. They used a myeloid-targeted STAT3 antisense oligonucleotide (CpG-STAT3ASO) in HNSCC in combination with TLR9 activation. This approach overcame resistance to radiotherapy in HNSCC mice regardless of HPV status. Combination treatment resulted in a reduction in residual M2 macrophages in the tumor and the recruitment of activated M1 macrophages to tumor-draining lymph nodes from irradiated tumors, thereby promoting antigen presentation and adaptive antitumor immune responses [212].

In a tumor mouse model, the activation of the macrophage TLR signaling pathway upregulated the expression of M1-type specific markers, such as MHC-II and co-stimulatory molecules (e.g., CD86, CD80, and CD40), thereby enhancing the phagocytosis and antitumor activity of macrophages. Imiquimod (TLR agonist) is the only FDA-approved topical treatment for squamous cell carcinoma.

Overexpression of the receptor for activated C kinase 1 (RACK1) is characteristic of HNSCC cells [213]. High RACK1 expression in HNSCC cells correlates with increased M2 macrophage infiltration in tumor samples. Moreover, the combination of RACK1 expression and the M2/M1 ratio could successfully predict prognosis in HNSCC [213]. The drug M435-1279, an inhibitor of the ubiquitin-conjugating enzyme E2T (UBE2T) that catalyzes RACK1 degradation, is currently being studied for its efficacy in HNSCC (but is being investigated in GC for now) [214].

## 11. TAMs and Soft Tissue Sarcomas

Soft tissue sarcomas (STSs) comprise heterogeneous rare tumors arising from connective tissues of mesenchymal origin. The etiology of STS remains understudied; however, some subtypes have been associated with exposure to environmental factors and genetic predisposition, including neurofibromatosis and Li–Fraumeni syndrome [215].

In a study by Dancsok et al. [216], in 960 patients with different subtypes of STSs, macrophages were predominant among immune cells in the TME. Also, Fan et al. [217] showed that in 472 patients with eight different sarcomas, the most abundant subpopulations in the TME were M2-phenotype macrophages (almost 34%) and non-activated (M0) macrophages (about 21%). This makes TAMs promising targets for the treatment of STS.

Pexidartinib, a CSF1 receptor inhibitor, is approved by the FDA for the treatment of adult patients with tenosynovial giant cell tumor, which is a rare and locally aggressive nonmalignant tumor that overexpresses CSF-1 [218,219,220,221]. Both in vitro and in vivo studies have demonstrated its ability to inhibit the proliferation of cells that are dependent on CSF1R stimulation and ligand-induced autophosphorylation of CSF1R. Pexidartinib administration decreased the number of M2 macrophages, with a concomitant decrease in Forkhead Box P3 (FOXP3)+ regulatory T lymphocytes, and increased CD8+ T lymphocyte migration [95].

The effect of zoledronic acid on decreasing TAMs in the TME has already been noted above in the text, but such effect was also noted in STS [222]. To overcome the short half-life of this drug, its conjugate with nanoparticles, CaZol@pMNP, was created. Injection of this conjugate into mice with sarcoma resulted in a decrease in the number of TAMs in the TME [96].

One promising strategy to alter macrophage polarization in tumors is the use of N-methyl-D-aspartate ion channel receptor (NMDAR) antagonists. RNA sequencing analysis of individual cells showed that blocking NMDAR promotes TAM repolarization in M1 macrophages in STS [115].

In testing a TLR2 agonist, the researchers were able to obtain macrophages with pronounced antitumor potential and achieve a significant increase in the M1/M2 ratio in mice with STS [223]. A synthetic agonist of Toll-like receptor 4 (TLR4), expressed by macrophages and other innate immune cells, is being tested in a phase I clinical trial for antitumor efficacy in patients with metastatic STS (NCT02180698) [224].

Activation of the CD47-SIRPα signaling cascade allows tumor cells to evade immune surveillance and suppress the phagocytic capacity of TAMs. Preclinical studies in mice with STS demonstrated the antitumor activity of TTI-621. TTI-621 has an affinity to SIRP1α and inhibits the binding of CD47 to SIRP1α, which induces the phagocytic activity of macrophages [100].

The targeted drug Regorafenib (an inhibitor of multiple protein kinases, including kinases involved in tumor angiogenesis (VEGFR1, −2, −3, TIE2), oncogenesis (KIT, RET, RAF-1, BRAF, BRAFv600E), metastasis (VEGFR3, PDGFR, FGFR), and the antitumor immune response (CSF1R)), prescribed for the treatment of STS, also has an effect on TAMs, providing repolarization in the direction of M1 macrophages [107]. The p38MAPK/Creb1/Klf4 signaling pathway may play a crucial role in Regorafenib-induced M2 to M1 polarization and their subsequent activation of T cells.

Trabectidine (a natural alkaloid derived from the Caribbean tunic), approved for the treatment of STS, in addition to its antitumor effect, has an effect on M2 TAMs: it causes double-strand DNA breaks in M2-TAMs, interrupting their cell cycle [98]. Also, Trabectidine can induce apoptosis of TAMs via TNF-associated apoptosis-inducing ligand (TRAIL) receptors, thereby selectively depleting monocytes or macrophages in blood and tumors.

## 12. Conclusions

TAMs play a key role in tumor progression in various types of malignancies. This makes them attractive as targets for antitumor therapy. Despite the large number of studies in this area, to date, there are no adequate approaches to antitumor therapy based on alterations in TAM functioning that would show high efficacy when administered in a mono-regimen for the treatment of malignant neoplasms. Studies devoted to the evaluation of the efficacy of drugs acting on TAMs are characterized by a small sample and the large heterogeneity of patient groups; in addition to this therapy, in such studies, chemotherapy or immunotherapy is used, which significantly complicates the evaluation of the effectiveness of the agent acting on TAMs. Thus, additional studies are needed to develop new approaches and improve the effectiveness of therapy aimed at targeting TAMs.

## Figures and Tables

**Table 1:** Impact on tumor-associated macrophages in different types of malignancies.

The Activation of Apoptosis of Macrophages in the Tumor and/or Their Exhaustion	
LC	One of the effects of Lurbinectedin prescribed for the treatment of metastatic SCLC causes selective apoptosis of TAMs [87].
BC	Liposome-conjugated zoledronic acid in triple-negative BC leads to TAM exhaustion [88]. DNA vaccine against asparaginyl endopeptidase legumin in mouse models resulted in TAM exhaustion [89]. Administration of the synthetic oleanane triterpenoid CDDO-methyl ester (CDDO-Me) to mice with BC resulted in TAM exhaustion [90]. Antibodies against CXCL12 resulted in the exhaustion of M2 TAMs [91]. Pharmacological inactivation by the selective PI3K p110δ inhibitor IC87114 in mouse models of BC caused a significant decrease in TAMs in tumor tissue [92].
CRC	In mouse models of CRC, treatment with RG7155 (a monoclonal antibody against CSF1R) reduced the amount of TAMs in the tumor [93].
GC	Bexmarilimab (FP-1305) is a humanized IgG4 monoclonal antibody specific to Clever-1 that has been shown to decrease the M2/M1 ratio following therapy [94]. The drug M435-1279, an inhibitor of the ubiquitin-conjugating enzyme E2T (UBE2T) that catalyzes RACK1 degradation, decreases the number of M2 macrophages.
STS	Pexidartinib decreases the number of M2 macrophages [95]. Calcium zoledronate conjugated to nanoparticles (CaZol@pMNP) promotes the reduction in TAMs [96]. AS16-Fc (anti-Ang-2/VEGF antibody) suppresses macrophage polarization toward M2 macrophages [97]. Trabectedin causes double-stranded DNA breaks in M2-TAMs, interrupting their cell cycle [98].
Increased phagocytic activity of macrophages	
LC	Human monoclonal antibodies that block the interaction between the programmed death receptor (PD-1) and its ligands (PD-L1 and PD-L2) reduce the phagocytic activity of TAMs in LC [99].
STS	TTI-621 has an affinity to SIRP1α and inhibits the binding of CD47 to SIRP1α, which induces phagocytic activity of macrophages [100].
Reprogramming of M2 macrophages into M1 macrophages	
LC	The inhibition of USP7 (plays a role as an oncoprotein) mediates TAM reprogramming into M1 through activation of the p38 MAPK pathway [101].
BC	Anemoside A3, which is an active compound from the perennial herbaceous plant Pulsatilla, activates macrophage polarization toward M1 through the TLR4/NF-κB/MAPK pathway, inhibiting the progression of BC [102]. Paclitaxel reprograms TAMs into M1 macrophages through the TLR4/NF-κB signaling cascade [103].
CRC	Cetuximab enhances the antitumor function of macrophages in an IL-6-dependent manner [104]. Maraviroc (CCR5 inhibitor) monotherapy in CRC resulted in the repolarization of macrophages toward an M1-like phenotype [105]. Cetuximab and panitumumab bind to the epidermal growth factor receptor (EGFR) and repolarize TAMs from M2-like phenotypes to M1-like phenotypes [106]. Regorafenib induces M2 TAM polarization to M1 macrophages [107].
PC	The use of nanoparticles targeting sialic acid with CSF-1R siRNA delivery leads to the reprogramming of macrophages into an M1 phenotype in human and mouse models of PC [108].
CC	Radiotherapy of cervical cancer induces an increase in the number of TAMs and a change in their subtype from the M2-like phenotype to the M1-like phenotype [109].
GC	mAb04-MICA (an antibody targeting VEGFR2 fused to the MICA α 1–α 2 ectodomain) induced the repolarization of TAMs from the M2 type to the M1 type both in vitro and in vivo [110]. CCX140-B (MedChemExpress, Monmouth Junction, NJ, USA) treatment, as a specific CCR2 inhibitor, efficiently blocked CCL2-CCR2 signaling, confirming the efficacy of CCL2 for TAMs [111]. Methionine enkephalin, an endogenous opioid pentapeptide, promotes the transition of TAMs from type M2 to type M1 [112]. Sophoridine promoted TAMs in gastric cancer to polarize toward the M1 type, as well as suppressed M2-type polarization [113].
HNSCC	The agonist STING (stimulator of interferon genes) in mouse models of PRGS exhibited the ability to reprogram TAMs from the M2 to M1 phenotype [114].
STS	NMDAR antagonists MK-801, memantine, and magnesium promote a change from a TAM phenotype to an antitumor phenotype [115]. Regorafenib induces M2 to M1 TAM polarization in STS [107].
CAR-M therapy	
BC	In the NCT04660929 clinical trial, anti-HER2 CAR-M demonstrated a favorable safety profile and early signs of antitumor activity in patients with HER2-overexpressing BC.
GC	In the NCT04660929 clinical trial, anti-HER2 CAR-M demonstrated a favorable safety profile and early signs of antitumor activity in patients with HER2-overexpressing GC. In the second clinical trial of NCT06224738, the efficacy of human HER2-CAR-M therapy for HER2-positive disseminated GC with metastasis to the peritoneum was evaluated.

Notes: tumor-associated macrophages—TAMs; LC—lung cancer; SCLC—small-cell lung cancer; BC—breast cancer; CRC—colorectal cancer; GC—gastric cancer; STS—soft tissue sarcomas; PC—prostate cancer; HNSCC—head and neck squamous cell cancer.

## Data Availability

No new data were created or analyzed in this study.

## References

[1] Gao J., Liang Y., Wang L. (2022). Shaping Polarization of Tumor-Associated Macrophages in Cancer Immunotherapy. Front. Immunol..

[2] Claasen H.H.V.R., Kluin P.M., Fleuren G.J. (1992). Tumor infiltrating cells in human cancer: On the possible role of CD16+ macrophages in antitumor cytotoxicity. Lab. Investig..

[3] Lin Y., Xu J., Lan H. (2019). Tumor-associated macrophages in tumor metastasis: Biological roles and clinical therapeutic applications. J. Hematol. Oncol..

[4] Van Dalen F.J., van Stevendaal M.H.M.E., Fennemann F.L., Verdoes M., Ilina O. (2019). Molecular repolarisation of tumour-associated macrophages. Molecules.

[5] Wang W., Liu W., Fidler T., Wang Y., Tang Y., Woods B., Welch C., Cai B., Silvestre-Roig C., Ai D. (2018). Macrophage inflammation, erythrophagocytosis, and accelerated atherosclerosis in JAK2V617F mice. Circ. Res..

[6] Biswas S.K., Mantovani A. (2010). Macrophage plasticity and interaction with lymphocyte subsets: Cancer as a paradigm. Nat. Immunol..

[7] West A.P., Brodsky I.E., Rahner C., Woo D.K., Erdjument-Bromage H., Tempst P., Walsh M.C., Choi Y., Shadel G.S., Ghosh S. (2011). TLR signalling augments macrophage bactericidal activity through mitochondrial ROS. Nature.

[8] Murray P.J., Allen J.E., Biswas S.K., Fisher E.A., Gilroy D.W., Goerdt S., Gordon S., Hamilton J.A., Ivashkiv L.B., Lawrence T. (2014). Macrophage Activation and Polarization: Nomenclature and Experimental Guidelines. Immunity.

[9] Zheng X., Turkowski K., Mora J., Brüne B., Seeger W., Weigert A., Savai R. (2017). Redirecting tumor-associated macrophages to become tumoricidal effectors as a novel strategy for cancer therapy. Oncotarget.

[10] Yao Y., Xu X.-H., Jin L. (2019). Macrophage polarization in physiological and pathological pregnancy. Front. Immunol..

[11] Mills C.D. (2012). M1 and M2 macrophages: Oracles of health and disease. Crit. Rev. Immunol..

[12] Honkanen T.J., Tikkanen A., Karihtala P., Mäkinen M., Väyrynen J.P., Koivunen J.P. (2019). Prognostic and predictive role of tumour-associated macrophages in HER2 positive breast cancer. Sci. Rep..

[13] Mantovani A., Sica A., Sozzani S., Allavena P., Vecchi A., Locati M. (2004). The chemokine system in diverse forms of macrophage activation and polarization. Trends Immunol..

[14] Mantovani A., Allavena P., Sica A. (2004). Tumour-associated macrophages as a prototypic type II polarised phagocyte population: Role in tumour progression. Eur. J. Cancer.

[15] Sica A., Schioppa T., Mantovani A., Allavena P. (2006). Tumour-associated macrophages are a distinct M2 polarised population promoting tumour progression: Potential targets of anti-cancer therapy. Eur. J. Cancer.

[16] Ng T.H.S., Britton G.J., Hill E.V., Verhagen J., Burton B.R., Wraith D.C. (2013). Regulation of adaptive immunity; the role of interleukin-10. Front. Immunol..

[17] Allavena P., Sica A., Garlanda C., Mantovani A. (2008). The Yin-Yang of tumor-associated macrophages in neoplastic progression and immune surveillance. Immunol. Rev..

[18] Condeelis J., Pollard J.W. (2006). Macrophages: Obligate partners for tumor cell migration, invasion, and metastasis. Cell.

[19] Qian B.Z., Pollard J.W. (2010). Macrophage Diversity Enhances Tumor Progression and Metastasis. Cell.

[20] Elchaninov A., Fatkhudinov T. MAKPOФAГИ. https://www.researchgate.net/publication/370940460_MAKROFAGI.

[21] Ma R.Y., Black A., Qian B.Z. (2022). Macrophage diversity in cancer revisited in the era of single-cell omics. Trends Immunol..

[22] Cheng S., Li Z., Gao R., Xing B., Gao Y., Yang Y., Qin S., Zhang L., Ouyang H., Du P. (2021). A pan-cancer single-cell transcriptional atlas of tumor infiltrating myeloid cells. Cell.

[23] Zilionis R., Engblom C., Pfirschke C., Savova V., Zemmour D., Saatcioglu H.D., Krishnan I., Maroni G., Meyerovitz C.V., Kerwin C.M. (2019). Single-Cell Transcriptomics of Human and Mouse Lung Cancers Reveals Conserved Myeloid Populations across Individuals and Species. Immunity.

[24] Mulder K., Patel A.A., Kong W.T., Piot C., Halitzk E., Dunsmore G., Khalilnezhad S., Irac S.E., Dubuisson A., Chevrier M. (2021). Cross-tissue single-cell landscape of human monocytes and macrophages in health and disease. Immunity.

[25] Gubin M.M., Esaulova E., Ward J.P., Malkova O.N., Runci D., Wong P., Noguchi T., Arthur C.D., Meng W., Alspach E. (2018). High-Dimensional Analysis Delineates Myeloid and Lymphoid Compartment Remodeling during Successful Immune-Checkpoint Cancer Therapy. Cell.

[26] Sadik A., Somarribas Patterson L.F., Öztürk S., Mohapatra S.R., Panitz V., Secker P.F., Pfänder P., Loth S., Salem H., Prentzell M.T. (2020). IL4I1 Is a Metabolic Immune Checkpoint that Activates the AHR and Promotes Tumor Progression. Cell.

[27] Molgora M., Esaulova E., Vermi W., Hou J., Chen Y., Luo J., Brioschi S., Bugatti M., Omodei A.S., Ricci B. (2020). TREM2 Modulation Remodels the Tumor Myeloid Landscape Enhancing Anti-PD-1 Immunotherapy. Cell.

[28] Katzenelenbogen Y., Sheban F., Yalin A., Yofe I., Svetlichnyy D., Jaitin D.A., Bornstein C., Moshe A., Keren-Shaul H., Cohen M. (2020). Coupled scRNA-Seq and Intracellular Protein Activity Reveal an Immunosuppressive Role of TREM2 in Cancer. Cell.

[29] Che L.H., Liu J.W., Huo J.P., Luo R., Xu R.M., He C., Li Y.Q., Zhou A.J., Huang P., Chen Y.Y. (2021). A single-cell atlas of liver metastases of colorectal cancer reveals reprogramming of the tumor microenvironment in response to preoperative chemotherapy. Cell Discov..

[30] Steele N.G., Carpenter E.S., Kemp S.B., Sirihorachai V.R., The S., Delrosario L., Lazarus J., Amir E.D., Gunchick V., Espinoza C. (2020). Multimodal mapping of the tumor and peripheral blood immune landscape in human pancreatic cancer. Nat. Cancer.

[31] Zhao Q., Kuang D.M., Wu Y., Xiao X., Li X.F., Li T.J., Zheng L. (2012). Activated CD69+ T Cells Foster Immune Privilege by Regulating IDO Expression in Tumor-Associated Macrophages. J. Immunol..

[32] Zhou Y., Yang D., Yang Q., Lv X., Huang W., Zhou Z., Wang Y., Zhang Z., Yuan T., Ding X. (2020). Single-cell RNA landscape of intratumoral heterogeneity and immunosuppressive microenvironment in advanced osteosarcoma. Nat. Commun..

[33] Zhang Q., Cheng S., Wang Y., Wang M., Lu Y., Wen Z., Ge Y., Ma Q., Chen Y., Zhang Y. (2021). Interrogation of the microenvironmental landscape in spinal ependymomas reveals dual functions of tumor-associated macrophages. Nat. Commun..

[34] Yin H., Guo R., Zhang H., Liu S., Gong Y., Yuan Y. (2021). A Dynamic Transcriptome Map of Different Tissue Microenvironment Cells Identified During Gastric Cancer Development Using Single-Cell RNA Sequencing. Front. Immunol..

[35] Griffith J.W., Sokol C.L., Luster A.D. (2014). Chemokines and chemokine receptors: Positioning cells for host defense and immunity. Annu. Rev. Immunol..

[36] Nagarsheth N., Wicha S.M., Zou W. (2017). Chemokines in the cancer microenvironment and their relevance in cancer immunotherapy. Nat. Rev. Immunol..

[37] Sharma A., Seow J.J.W., Dutertre C.A., Pai R., Blériot C., Mishra A., Wong R.M.M., Singh G.S.N., Sudhagar S., Khalilnezhad S. (2020). Onco-fetal Reprogramming of Endothelial Cells Drives Immunosuppressive Macrophages in Hepatocellular Carcinoma. Cell.

[38] Sathe A., Grimes S.M., Lau B.T., Chen J., Suarez C., Huang R.J., Poultsides G., Ji H.P. (2020). Single-Cell Genomic Characterization Reveals the Cellular Reprogramming of the Gastric Tumor Microenvironment. Clin. Cancer Res..

[39] Zhang P., Yang M., Zhang Y., Xiao S., Lai X., Tan A., Du S., Li S. (2019). Dissecting the Single-Cell Transcriptome Network Underlying Gastric Premalignant Lesions and Early Gastric Cancer. Cell Rep..

[40] Di Conza G., Tsai C.H., Gallart-Ayala H., Yu Y.R., Franco F., Zaffalon L., Xie X., Li X., Xiao Z., Raines L.N. (2021). Tumor-induced reshuffling of lipid composition on the endoplasmic reticulum membrane sustains macrophage survival and pro-tumorigenic activity. Nat. Immunol..

[41] Zhang L., Li Z., Skrzypczynska K.M., Fang Q., Zhang W., O’Brien S.A., He Y., Wang L., Zhang Q., Kim A. (2020). Single-Cell Analyses Inform Mechanisms of Myeloid-Targeted Therapies in Colon Cancer. Cell.

[42] Talks K.L., Turley H., Gatter K.C., Maxwell P.H., Pugh C.W., Ratcliffe P.J., Harris A.L. (2000). The expression and distribution of the hypoxia-inducible factors HIF-1α and HIF-2α in normal human tissues, cancers, and tumor-associated macrophages. Am. J. Pathol..

[43] Pombo Antunes A.R., Scheyltjens I., Lodi F., Messiaen J., Antoranz A., Duerinck J., Kancheva D., Martens L., De Vlaminck K., Van Hove H. (2021). Single-cell profiling of myeloid cells in glioblastoma across species and disease stage reveals macrophage competition and specialization. Nat. Neurosci..

[44] Harney A.S., Arwert E.N., Entenberg D., Wang Y., Guo P., Qian B.Z., Oktay M.H., Pollard J.W., Jones J.G., Condeelis J.S. (2015). Real-time imaging reveals local, transient vascular permeability, and tumor cell intravasation stimulated by TIE2hi macrophage–derived VEGFA. Cancer Discov..

[45] Hughes R., Qian B.Z., Rowan C., Muthana M., Keklikoglou I., Olson O.C., Tazzyman S., Danson S., Addison C., Clemons M. (2015). Perivascular M2 macrophages stimulate tumor relapse after chemotherapy. Cancer Res..

[46] Casanova-Acebes M., Dalla E., Leader A.M., LeBerichel J., Nikolic J., Morales B.M., Brown M., Chang C., Troncoso L., Chen S.T. (2021). Tissue-resident macrophages provide a pro-tumorigenic niche to early NSCLC cells. Nature.

[47] Massalha H., Bahar Halpern K., Abu-Gazala S., Jana T., Massasa E.E., Moor A.E., Buchauer L., Rozenberg M., Pikarsky E., Amit I. (2020). A single cell atlas of the human liver tumor microenvironment. Mol. Syst. Biol..

[48] Kim N., Kim H.K., Lee K., Hong Y., Cho J.H., Choi J.W., Lee J.I., Suh Y.L., Ku B.M., Eum H.H. (2020). Single-cell RNA sequencing demonstrates the molecular and cellular reprogramming of metastatic lung adenocarcinoma. Nat. Commun..

[49] Hara T., Chanoch-Myers R., Mathewson N.D., Myskiw C., Atta L., Bussema L., Eichhorn S.W., Greenwald A.C., Kinker G.S., Rodman C. (2021). Interactions between cancer cells and immune cells drive transitions to mesenchymal-like states in glioblastoma. Cancer Cell.

[50] Tuong Z.K., Loudon K.W., Berry B., Richoz N., Jones J., Tan X., Nguyen Q., George A., Hori S., Field S. (2021). Resolving the immune landscape of human prostate at a single-cell level in health and cancer. Cell Rep..

[51] Wang J., Zhu N., Su X., Gao Y., Yang R. (2023). Novel tumor-associated macrophage populations and subpopulations by single cell RNA sequencing. Front. Immunol..

[52] Quail D.F., Joyce J.A. (2013). Microenvironmental regulation of tumor progression and metastasis. Nat. Med..

[53] Lewis C.E., Pollard J.W. (2006). Distinct role of macrophages in different tumor microenvironments. Cancer Res..

[54] Locati M., Curtale G., Mantovani A. (2020). Diversity, Mechanisms, and Significance of Macrophage Plasticity. Annu. Rev. Pathol. Mech. Dis..

[55] Cassetta L., Kitamura T. (2018). Macrophage targeting: Opening new possibilities for cancer immunotherapy. Immunology.

[56] Lechien J.R., Descamps G., Seminerio I., Furgiuele S., Dequanter D., Mouawad F., Badoual C., Journe F., Saussez S. (2020). HPV involvement in the tumor microenvironment and immune treatment in head and neck squamous cell carcinomas. Cancers.

[57] Mhaidly N., Journe F., Najem A., Stock L., Trelcat A., Dequanter D., Saussez S., Descamps G. (2023). Macrophage Profiling in Head and Neck Cancer to Improve Patient Prognosis and Assessment of Cancer Cell–Macrophage Interactions Using Three-Dimensional Coculture Models. Int. J. Mol. Sci..

[58] Nywening T.M., Belt B.A., Cullinan D.R., Panni R.Z., Han B.J., Sanford D.E., Jacobs R.C., Ye J., Patel A.A., Gillanders W.E. (2017). Targeting both tumour-associated CXCR2+ neutrophils and CCR2+ macrophages disrupts myeloid recruitment and improves chemotherapeutic responses in pancreatic ductal adenocarcinoma. Gut.

[59] Dammeijer F., Lievense L.A., Kaijen-Lambers M.E., van Nimwegen M., Bezemer K., Hegmans J.P., van Hall T., Hendriks R.W., Aerts J.G. (2017). Depletion of tumor-associated macrophages with a CSF-1R kinase inhibitor enhances antitumor immunity and survival induced by DC immunotherapy. Cancer Immunol. Res..

[60] Bart V.M.T., Pickering R.J., Taylor P.R., Ipseiz N. (2021). Macrophage reprogramming for therapy. Immunology.

[61] Weiskopf K. (2017). Cancer immunotherapy targeting the CD47/SIRPα axis. Eur. J. Cancer.

[62] Mantovani A., Marchesi F., Malesci A., Laghi L., Allavena P. (2017). Tumour-associated macrophages as treatment targets in oncology. Nat. Rev. Clin. Oncol..

[63] Klichinsky M., Ruella M., Shestova O., Lu X.M., Best A., Zeeman M., Schmierer M., Gabrusiewicz K., Anderson N.R., Petty N.E. (2020). Human chimeric antigen receptor macrophages for cancer immunotherapy. Nat. Biotechnol..

[64] Villanueva M.T. (2020). Macrophages get a CAR. Nat. Rev. Cancer.

[65] Huang T., Bei C., Hu Z., Li Y. (2024). CAR-macrophage: Breaking new ground in cellular immunotherapy. Front. Cell Dev. Biol..

[66] Qian B.Z., Li J., Zhang H., Kitamura T., Zhang J., Campion L.R., Kaiser E.A., Snyder L.A., Pollard J.W. (2011). CCL2 recruits inflammatory monocytes to facilitate breast-tumour metastasis. Nature.

[67] Ren G., Zhao X., Wang Y., Zhang X., Chen X., Xu C., Yuan Z.R., Roberts A.I., Zhang L., Zheng B. (2012). CCR2-dependent recruitment of macrophages by tumor-educated mesenchymal stromal cells promotes tumor development and is mimicked by TNFα. Cell Stem Cell.

[68] Pathria P., Louis T.L., Varner J.A. (2019). Targeting Tumor-Associated Macrophages in Cancer. Trends Immunol..

[69] Guerriero J.L. (2018). Macrophages: The Road Less Traveled, Changing Anticancer Therapy. Trends Mol. Med..

[70] Nywening T.M., Wang-Gillam A., Sanford D.E., Belt B.A., Panni R.Z., Cusworth B.M., Toriola A.T., Nieman R.K., Worley L.A., Yano M. (2016). Targeting tumour-associated macrophages with CCR2 inhibition in combination with FOLFIRINOX in patients with borderline resectable and locally advanced pancreatic cancer: A single-centre, open-label, dose-finding, non-randomised, phase 1b trial. Lancet Oncol..

[71] Teicher B.A., Fricker S.P. (2010). CXCL12 (SDF-1)/CXCR4 pathway in cancer. Clin. Cancer Res..

[72] Scala S. (2015). Molecular pathways: Targeting the CXCR4-CXCL12 Axis-Untapped potential in the tumor microenvironment. Clin. Cancer Res..

[73] Hume D.A., MacDonald K.P.A. (2012). Therapeutic applications of macrophage colony-stimulating factor-1 (CSF-1) and antagonists of CSF-1 receptor (CSF-1R) signaling. Blood.

[74] Peyraud F., Cousin S., Italiano A. (2017). CSF-1R Inhibitor Development: Current Clinical Status. Curr. Oncol. Rep..

[75] Zhu Y., Knolhoff B.L., Meyer M.A., Nywening T.M., West B.L., Luo J., Wang-Gillam A., Goedegebuure S.P., Linehan D.C., DeNardo D.G. (2014). CSF1/CSF1R blockade reprograms tumor-infiltrating macrophages and improves response to T-cell checkpoint immunotherapy in pancreatic cancer models. Cancer Res..

[76] Falchook G.S., Peeters M., Rottey S., Dirix L.Y., Obermannova R., Cohen J.E., Perets R., Frommer R.S., Bauer T.M., Wang J.S. (2021). A phase 1a/1b trial of CSF-1R inhibitor LY3022855 in combination with durvalumab or tremelimumab in patients with advanced solid tumors. Investig. New Drugs.

[77] Tolcher A.W., Rasco D., Sharma S., Taylor M., Quaranto C., Tamang D.L., Nordness R., Meyers M.L., Sankoh S., Ordentlich P. (2020). Abstract CT242: SNDX-6352-0502: A phase 1, open-label, dose escalation trial to investigate the safety, tolerability, pharmacokinetics and pharmacodynamic activity of SNDX-6352 in combination with durvalumab in patients with unresectable, recurrent, locally-advanced, or metastatic solid tumors. Cancer Res..

[78] Kuemmel S., Campone M., Loirat D., Lopez R.L., Beck J.T., De Laurentiis M., Im S.A., Kim S.B., Kwong A., Steger G.G. (2022). A Randomized Phase II Study of Anti-CSF1 Monoclonal Antibody Lacnotuzumab (MCS110) Combined with Gemcitabine and Carboplatin in Advanced Triple-Negative Breast Cancer. Clin. Cancer Res..

[79] Wainberg Z., Piha-Paul S.A., Luke J., Kim E.J., Thompson J.A., Britten C.D., Johnson J.M., Pfanzelter N., Gordon M., Rasco D.W. (2017). First-in-human phase 1 dose escalation and expansion of a novel combination, anti-CSF-1 receptor (cabiralizumab) plus anti-PD-1 (nivolumab), in patients with advanced solid tumors. J. Immunother. Cancer.

[80] Benner B., Good L., Quiroga D., Schultz T.E., Kassem M., Carson W.E., Cherian M.A., Sardesai S., Wesolowski R. (2020). Pexidartinib, a novel small molecule csf-1r inhibitor in use for tenosynovial giant cell tumor: A systematic review of pre-clinical and clinical development. Drug Des. Dev. Ther..

[81] Saung M.T., Muth S., Ding D., Thomas D.L., Blair A.B., Tsujikawa T., Coussens L., Jaffee E.M., Zheng L. (2018). Targeting myeloid-inflamed tumor with anti-CSF-1R antibody expands CD137+ effector T-cells in the murine model of pancreatic cancer. J. Immunother. Cancer.

[82] Di Caro G., Cortese N., Castino G.F., Grizzi F., Gavazzi F., Ridolfi C., Capretti G., Mineri R., Todoric J., Zerbi A. (2015). Dual prognostic significance of tumour-Associated macrophages in human pancreatic adenocarcinoma treated or untreated with chemotherapy. Gut.

[83] Malesci A., Bianchi P., Celesti G., Basso G., Marchesi F., Grizzi F., Di Caro G., Cavalleri T., Rimassa L., Palmqvist R. (2017). Tumor-associated macrophages and response to 5-fluorouracil adjuvant therapy in stage III colorectal cancer. OncoImmunology.

[84] Heath O., Berlato C., Maniati E., Lakhani A., Pegrum C., Kotantaki P., Elorbany S., Böhm S., Barry S.T., Annibaldi A. (2021). Chemotherapy induces tumor-associated macrophages that aid adaptive immune responses in ovarian cancer. Cancer Immunol. Res..

[85] Florido J., Martinez-Ruiz L., Rodriguez-Santana C., López-Rodríguez A., Hidalgo-Gutiérrez A., Cottet-Rousselle C., Lamarche F., Schlattner U., Guerra-Librero A., Aranda-Martínez P. (2022). Melatonin drives apoptosis in head and neck cancer by increasing mitochondrial ROS generated via reverse electron transport. J. Pineal Res..

[86] Rodriguez-Garcia A., Lynn R.C., Poussin M., Eiva M.A., Shaw L.C., O’Connor R.S., Minutolo N.G., Casado-Medrano V., Lopez G., Matsuyama T. (2021). CAR-T cell-mediated depletion of immunosuppressive tumor-associated macrophages promotes endogenous antitumor immunity and augments adoptive immunotherapy. Nat. Commun..

[87] Allavena P., Belgiovine C., Digifico E., Frapolli R., D’Incalci M. (2022). Effects of the Anti-Tumor Agents Trabectedin and Lurbinectedin on Immune Cells of the Tumor Microenvironment. Front. Oncol..

[88] Cai X.J., Wang Z., Cao J.W., Ni J.J., Xu Y.Y., Yao J., Xu H., Liu F., Yang G.Y. (2017). Anti-angiogenic and anti-tumor effects of metronomic use of novel liposomal zoledronic acid depletes tumor-associated macrophages in triple negative breast cancer. Oncotarget.

[89] Lewen S., Zhou H., Hu H.D., Cheng T., Markowitz D., Reisfeld R.A., Xiang R., Luo Y. (2008). A Legumain-based minigene vaccine targets the tumor stroma and suppresses breast cancer growth and angiogenesis. Cancer Immunol. Immunother..

[90] Ball M.S., Bhandari R., Torres G.M., Martyanov V., ElTanbouly M.A., Archambault K., Whitfield M.L., Liby K.T., Pioli P.A. (2020). CDDO-Me Alters the Tumor Microenvironment in Estrogen Receptor Negative Breast Cancer. Sci. Rep..

[91] Seoane S., Martinez-Ordoñez A., Eiro N., Cabezas-Sainz P., Garcia-Caballero L., Gonzalez L.O., Macia M., Sanchez L., Vizoso F., Perez-Fernandez R. (2019). POU1F1 transcription factor promotes breast cancer metastasis via recruitment and polarization of macrophages. J. Pathol..

[92] Goulielmaki E., Bermudez-Brito M., Andreou M., Tzenaki N., Tzardi M., de Bree E., Tsentelierou E., Makrigiannakis A., Papakonstanti E.A. (2018). Pharmacological inactivation of the PI3K p110δ prevents breast tumour progression by targeting cancer cells and macrophages article. Cell Death Dis..

[93] Ries C.H., Cannarile M.A., Hoves S., Benz J., Wartha K., Runza V., Rey-Giraud F., Pradel L.P., Feuerhake F., Klaman I. (2014). Targeting tumor-associated macrophages with anti-CSF-1R antibody reveals a strategy for cancer therapy. Cancer Cell.

[94] Rannikko J.H., Verlingue L., de Miguel M., Pasanen A., Robbrecht D., Skytta T., Iivanainen S., Shetty S., Ma Y.T., Graham D.M. (2023). Bexmarilimab-induced macrophage activation leads to treatment benefit in solid tumors: The phase I/II first-in-human MATINS trial. Cell Rep. Med..

[95] Fujiwara T., Yakoub M.A., Chandler A., Christ A.B., Yang G., Ouerfelli O., Rajasekhar V.K., Yoshida A., Kondo H., Hata T. (2021). CSF1/CSF1 R signaling inhibitor pexidartinib (PLX3397) reprograms tumor-associated macrophages and stimulates T-cell infiltration in the sarcoma microenvironment. Mol. Cancer Ther..

[96] Zang X., Zhang X., Hu H., Qiao M., Zhao X., Deng Y., Chen D. (2019). Targeted Delivery of Zoledronate to Tumor-Associated Macrophages for Cancer Immunotherapy. Mol. Pharm..

[97] Zhu X., Yang J., Gao Y., Wu C., Yi L., Li G., Qi Y. (2018). The dual effects of a novel peptibody on angiogenesis inhibition and M2 macrophage polarization on sarcoma. Cancer Lett..

[98] Gordon E.M., Sankhala K.K., Chawla N., Chawla S.P. (2016). Trabectedin for Soft Tissue Sarcoma: Current Status and Future Perspectives. Adv. Ther..

[99] Ma H., Zhang Z., Hu Q., Chen H., Wu G., Zhou Y., Xue Q. (2023). Shedding light on macrophage immunotherapy in lung cancer. J. Cancer Res. Clin. Oncol..

[100] Ansell S., Maris M.B., Lesokhin A.M., Chen R.W., Flinn I.W., Sawas A., Minden M.D., Villa D., Percival M.M., Advani A.S. (2016). A Phase 1 Study of TTI-621, a Novel Immune Checkpoint Inhibitor Targeting CD47, in Patients with Relapsed or Refractory Hematologic Malignancies. Blood.

[101] Dai X., Lu L., Deng S., Meng J., Wan C., Huang J., Sun Y., Hu Y., Wu B., Wu G. (2020). USP7 targeting modulates anti-tumor immune response by reprogramming Tumor-associated Macrophages in Lung Cancer. Theranostics.

[102] Yin L., Fan Z., Liu P., Chen L., Guan Z., Liu Y., Luo Y. (2021). Anemoside A3 activates TLR4-dependent M1-phenotype macrophage polarization to represses breast tumor growth and angiogenesis. Toxicol. Appl. Pharmacol..

[103] Wanderley C.W., Colón D.F., Luiz J.P.M., Oliveira F.F., Viacava P.R., Leite C.A., Pereira J.A., Silva C.M., Silva C.R., Silva R.L. (2018). Paclitaxel reduces tumor growth by reprogramming tumor-associated macrophages to an M1 profile in a TLR4-dependent manner. Cancer Res..

[104] Zhao Y., Liu X., Huo M., Wang Y., Li Y., Xu N., Zhu H. (2020). Cetuximab enhances the anti-tumor function of macrophages in an IL-6 dependent manner. Life Sci..

[105] Suarez-Carmona M., Chaorentong P., Kather J.N., Rothenheber R., Ahmed A., Berthel A., Heinzelmann A., Moraleda R., Valous N.A., Kosaloglu Z. (2019). CCR5 status and metastatic progression in colorectal cancer. OncoImmunology.

[106] Taniguchi H., Yamanaka T., Sakai D., Muro K., Yamazaki K., Nakata S., Kimura H., Ruff P., Kim T.W., Peeters M. (2020). Efficacy of panitumumab and cetuximab in patients with colorectal cancer previously treated with bevacizumab; a combined analysis of individual patient data from aspecct and wjog6510g. Cancers.

[107] Ou D.L., Chen C.W., Hsu C.L., Chung C.H., Feng Z.R., Lee B.S., Cheng A.L., Yang M.H., Hsu C. (2021). Regorafenib enhances antitumor immunity via inhibition of p38 kinase/Creb1/Klf4 axis in tumor-associated macrophages. J. Immunother. Cancer.

[108] Sun Y., Cronin M.F., Mendonça M.C.P., Guo J., O’Driscoll C.M. (2023). Sialic acid-targeted cyclodextrin-based nanoparticles deliver CSF-1R siRNA and reprogram tumour-associated macrophages for immunotherapy of prostate cancer. Eur. J. Pharm. Sci..

[109] Ren J., Li L., Yu B., Xu E., Sun N., Li X., Xing Z., Han X., Cui Y., Wang X. (2022). Extracellular vesicles mediated proinflammatory macrophage phenotype induced by radiotherapy in cervical cancer. BMC Cancer.

[110] Pan M., Wang F., Nan L., Yang S., Qi J., Xie J., Shao S., Zou H., Wang M., Sun F. (2023). αVEGFR2-MICA fusion antibodies enhance immunotherapy effect and synergize with PD-1 blockade. Cancer Immunol. Immunother..

[111] Sun W., Wang X., Wang D., Lu L., Lin H., Zhang Z., Jia Y., Nie X., Liu T., Fu W. (2022). CD40×HER2 bispecific antibody overcomes the CCL2-induced trastuzumab resistance in HER2-positive gastric cancer. J. Immunother. Cancer.

[112] Wang X., Jiao X., Meng Y., Chen H., Griffin N., Gao X., Shan F. (2018). Methionine enkephalin (MENK) inhibits human gastric cancer through regulating tumor associated macrophages (TAMs) and PI3K/AKT/mTOR signaling pathway inside cancer cells. Int. Immunopharmacol..

[113] Zhuang H., Dai X., Zhang X., Mao Z., Huang H. (2020). Sophoridine suppresses macrophage-mediated immunosuppression through TLR4/IRF3 pathway and subsequently upregulates CD8+ T cytotoxic function against gastric cancer. Biomed. Pharmacother..

[114] Wu Y.T., Fang Y., Wei Q., Shi H., Tan H., Deng Y., Zeng Z., Qiu J., Chen C., Sun L. (2022). Tumor-targeted delivery of a STING agonist improves cancer immunotherapy. Proc. Natl. Acad. Sci. USA.

[115] Yuan D., Hu J., Ju X., Putz E.M. (2023). NMDAR antagonists suppress tumor progression by regulating tumor-associated macrophages. Proc. Natl. Acad. Sci. USA.

[116] Inamura K. (2017). Lung cancer: Understanding its molecular pathology and the 2015 wHO classification. Front. Oncol..

[117] Alduais Y., Zhang H., Fan F., Chen J., Chen B. (2023). Non-small cell lung cancer (NSCLC): A review of risk factors, diagnosis, and treatment. Medicine.

[118] Bernabé-Caro R., Chen Y., Dowlati A., Eason P. (2023). Current and Emerging Treatment Options for Patients with Relapsed Small-cell Lung Carcinoma: A Systematic Literature Review. Clin. Lung Cancer.

[119] Massuti B., Cobo M., Camps C., Dómine M., Provencio M., Alberola V., Viñolas N., Rosell R., Tarón M., Gutiérrez-Calderón V. (2012). Trabectedin in patients with advanced non-small-cell lung cancer (NSCLC) with XPG and/or ERCC1 overexpression and BRCA1 underexpression and pretreated with platinum. Lung Cancer.

[120] Sedighzadeh S.S., Khoshbin A.P., Razi S., Keshavarz-Fathi M., Rezaei N. (2021). A narrative review of tumor-associated macrophages in lung cancer: Regulation of macrophage polarization and therapeutic implications. Transl. Lung Cancer Res..

[121] Liu L., Chen G., Gong S., Huang R., Fan C. (2023). Targeting tumor-associated macrophage: An adjuvant strategy for lung cancer therapy. Front. Immunol..

[122] Wang R., Liu Z., Wang T., Zhang J., Liu J., Zhou Q. (2024). Landscape of adenosine pathway and immune checkpoint dual blockade in NSCLC: Progress in basic research and clinical application. Front. Immunol..

[123] Zhang W., Huang Q., Xiao W., Zhao Y., Pi J., Xu H., Zhao H., Xu J., Evans C.E., Jin H. (2020). Advances in Anti-Tumor Treatments Targeting the CD47/SIRPα Axis. Front. Immunol..

[124] Yang Z., Peng Y., Guo W., Xu J., Li L., Tian H., Li R., Liu L., Tan F., Gao S. (2021). PD-L1 and CD47 co-expression predicts survival and enlightens future dual-targeting immunotherapy in non-small cell lung cancer. Thorac. Cancer.

[125] Wang R., Lu M., Zhang J., Chen S., Luo X., Qin Y., Chen H. (2011). Increased IL-10 mRNA expression in tumor-associated macrophage correlated with late stage of lung cancer. J. Exp. Clin. Cancer Res..

[126] Yang L., Yang L., Dong Y., Li Y., Wang D., Liu S., Wang D., Gao Q., Ji S., Chen X. (2019). IL-10 derived from M2 macrophage promotes cancer stemness via JAK1/STAT1/NF-κB/Notch1 pathway in non-small cell lung cancer. Int. J. Cancer.

[127] La Fleur L., Botling J., He F., Pelicano C., Zhou C., He C., Palano G., Mezheyeuski A., Micke P., Ravetch J.V. (2021). Targeting MARCO and IL37R on immunosuppressive macrophages in lung cancer blocks regulatory T cells and supports cytotoxic lymphocyte function. Cancer Res..

[128] Xu H., Xu B. (2023). Breast cancer: Epidemiology, risk factors and screening. Chin. J. Cancer Res..

[129] Admoun C., Mayrovitz H.N. (2022). The Etiology of Breast Cancer. Breast Cancer.

[130] Zhang Q.W., Liu L., Gong C.Y., Shi H.S., Zeng Y.H., Wang X.Z., Zhao Y.W., Wei Y.Q. (2012). Prognostic Significance of Tumor-Associated Macrophages in Solid Tumor: A Meta-Analysis of the Literature. PLoS ONE.

[131] Komohara Y., Kurotaki D., Tsukamoto H., Miyasato Y., Yano H., Pan C., Yamamoto Y., Fujiwara Y. (2023). Involvement of protumor macrophages in breast cancer progression and characterization of macrophage phenotypes. Cancer Sci..

[132] Larionova I., Tuguzbaeva G., Ponomaryova A., Stakheyeva M., Cherdyntseva N., Pavlov V., Choinzonov E., Kzhyshkowska J. (2020). Tumor-Associated Macrophages in Human Breast, Colorectal, Lung, Ovarian and Prostate Cancers. Front. Oncol..

[133] Tiainen S., Tumelius R., Rilla K., Hämäläinen K., Tammi M., Tammi R., Kosma V.M., Oikari S., Auvinen P. (2015). High numbers of macrophages, especially M2-like (CD163-positive), correlate with hyaluronan accumulation and poor outcome in breast cancer. Histopathology.

[134] Zhang W.-J., Wang X.H., Gao S.T., Chen C., Xu X.Y., Sun Q., Zhou Z.H., Wu G.Z., Yu Q., Xu G. (2018). Tumor-associated macrophages correlate with phenomenon of epithelial-mesenchymal transition and contribute to poor prognosis in triple-negative breast cancer patients. J. Surg. Res..

[135] Carron E.C., Homra S., Rosenberg J., Coffelt S.B., Kittrell F., Zhang Y., Creighton C.J., Fuqua S.A., Medina D., Machado H.L. (2017). Macrophages promote the progression of premalignant mammary lesions to invasive cancer. Oncotarget.

[136] Khan S.U., Khan M.U., Azhar Ud Din M., Khan I.M., Khan M.I., Bungau S., Hassan S.S.U. (2023). Reprogramming tumor-associated macrophages as a unique approach to target tumor immunotherapy. Front. Immunol..

[137] Muteeb G., Khafaga D.S., El-Morsy M.T., Farhan M., Aatif M., Hosney M. (2024). Targeting tumor-associated macrophages with nanocarrier-based treatment for breast cancer: A step toward developing innovative anti-cancer therapeutics. Heliyon.

[138] Niu X., Ma J., Li J., Gu Y., Yin L., Wang Y., Zhou X., Wang J., Ji H., Zhang Q. (2021). Sodium/glucose cotransporter 1-dependent metabolic alterations induce tamoxifen resistance in breast cancer by promoting macrophage M2 polarization. Cell Death Dis..

[139] Yang C., He L., He P., Liu Y., Wang W., He Y., Du Y., Gao F. (2015). Increased drug resistance in breast cancer by tumor-associated macrophages through IL-10/STAT3/bcl-2 signaling pathway. Med. Oncol..

[140] Xiao M., He J., Yin L., Chen X., Zu X., Shen Y. (2021). Tumor-Associated Macrophages: Critical Players in Drug Resistance of Breast Cancer. Front. Immunol..

[141] Tamimi R.M., Brugge J.S., Freedman M.L., Miron A., Iglehart J.D., Colditz G.A., Hankinson S.E. (2008). Circulating colony stimulating factor-1 and breast cancer risk. Cancer Res..

[142] Aharinejad S., Paulus P., Sioud M., Hofmann M., Zins K., Schäfer R., Stanley E.R., Abraham D. (2004). Colony-stimulating factor-1 blockade by antisense oligonucleotides and small interfering RNAs suppresses growth of human mammary tumor xenografts in mice. Cancer Res..

[143] Zhao Z., Zheng L., Chen W., Weng W., Song J., Ji J. (2019). Delivery strategies of cancer immunotherapy: Recent advances and future perspectives. J. Hematol. Oncol..

[144] Gomez-Roca C.A., Italiano A., Le Tourneau C., Cassier P.A., Toulmonde M., D’Angelo S.P., Campone M., Weber K.L., Loirat D., Cannarile M.A. (2019). Phase i study of emactuzumab single agent or in combination with paclitaxel in patients with advanced/metastatic solid tumors reveals depletion of immunosuppressive M2-like macrophages. Ann. Oncol..

[145] MacHiels J.P., Gomez-Roca C., Michot J.M., Zamarin D., Mitchell T., Catala G., Eberst L., Jacob W., Jegg A.M., Cannarile M.A. (2020). Phase Ib study of anti-CSF-1R antibody emactuzumab in combination with CD40 agonist selicrelumab in advanced solid tumor patients. J. Immunother. Cancer.

[146] Shang L., Zhong Y., Yao Y., Liu C., Wang L., Zhang W., Liu J., Wang X., Sun C. (2023). Subverted macrophages in the triple-negative breast cancer ecosystem. Biomed. Pharmacother..

[147] Zielińska K.A., Katanaev V.L. (2020). The signaling duo CXCL12 and CXCR4: Chemokine fuel for breast cancer tumorigenesis. Cancers.

[148] Kuninty P.R., Schnittert J., Storm G., Prakash J. (2016). MicroRNA targeting to modulate tumor microenvironment. Front. Oncol..

[149] Wang W., Liu Y., Guo J., He H., Mi X., Chen C., Xie J., Wang S., Wu P., Cao F. (2018). miR-100 maintains phenotype of tumor-associated macrophages by targeting mTOR to promote tumor metastasis via Stat5a/IL-1ra pathway in mouse breast cancer. Oncogenesis.

[150] Junankar S., Shay G., Jurczyluk J., Ali N., Down J., Pocock N., Parker A., Nguyen A., Sun S., Kashemirov B. (2015). Real-time intravital imaging establishes tumor-associated macrophages as the extraskeletal target of bisphosphonate action in cancer. Cancer Discov..

[151] Zollo M., Di Dato V., Spano D., De Martino D., Liguori L., Marino N., Vastolo V., Navas L., Garrone B., Mangano G. (2012). Targeting monocyte chemotactic protein-1 synthesis with bindarit induces tumor regression in prostate and breast cancer animal models. Clin. Exp. Metastasis.

[152] Sung H., Ferlay J., Siegel R.L., Laversanne M., Soerjomataram I., Jemal A., Bray F. (2021). Global Cancer Statistics 2020: GLOBOCAN Estimates of Incidence and Mortality Worldwide for 36 Cancers in 185 Countries. CA Cancer J. Clin..

[153] Kanth P., Grimmett J., Champine M., Burt R., Samadder N.J. (2017). Hereditary Colorectal Polyposis and Cancer Syndromes: A Primer on Diagnosis and Management. Am. J. Gastroenterol..

[154] Liu Z. (2019). Gastrointestinal Cancers.

[155] Kang J.C., Chen J.S., Lee C.H., Chang J.J., Shieh Y.S. (2010). Intratumoral macrophage counts correlate with tumor progression in colorectal cancer. J. Surg. Oncol..

[156] Kwak Y., Koh J., Kim D.W., Kang S.B., Kim W.H., Lee H.S. (2016). Immunoscore encompassing CD3+ and CD8+ T cell densities in distant metastasis is a robust prognostic marker for advanced colorectal cancer. Oncotarget.

[157] Forssell J., Öberg Å., Henriksson M.L., Stenling R., Jung A., Palmqvist R. (2007). High macrophage infiltration along the tumor front correlates with improved survival in colon cancer. Clin. Cancer Res..

[158] Zhou Q., Peng R.Q., Wu X.J., Xia Q., Hou J.H., Ding Y., Zhou Q.M., Zhang X., Pang Z.Z., Wan D.S. (2010). The density of macrophages in the invasive front is inversely correlated to liver metastasis in colon cancer. J. Transl. Med..

[159] Lan J., Sun L., Xu F., Liu L., Hu F., Song D., Hou Z., Wu W., Luo X., Wang J. (2019). M2 macrophage-derived exosomes promote cell migration and invasion in colon cancer. Cancer Res..

[160] Marech I., Ammendola M., Sacco R., Sammarco G., Zuccalà V., Zizzo N., Leporini C., Luposella M., Patruno R., Filippelli G. (2016). Tumour-associated macrophages correlate with microvascular bed extension in colorectal cancer patients. J. Cell. Mol. Med..

[161] Tacconi C., Ungaro F., Correale C., Arena V., Massimino L., Detmar M., Spinelli A., Carvello M., Mazzone M., Oliveira A.I. (2019). Activation of the VEGFC/VEGFR3 pathway induces tumor immune escape in colorectal cancer. Cancer Res..

[162] Xu H., Zhang Y., Peña M.M., Pirisi L., Creek K.E. (2017). Six1 promotes colorectal cancer growth and metastasis by stimulating angiogenesis and recruiting tumorassociated macrophages. Carcinogenesis.

[163] Song W., Ma J., Lei B., Yuan X., Cheng B., Yang H., Wang M., Feng Z., Wang L. (2018). Sine oculis homeobox 1 promotes proliferation and migration of human colorectal cancer cells through activation of Wnt/β-catenin signaling. Cancer Sci..

[164] Razak A.R.A., Cleary J.M., Moreno V., Boyer M., Calvo Aller E., Edenfield W., Tie J., Harvey R.D., Rutten A., Shah M.A. (2020). Safety and efficacy of AMG 820, an anti-colony-stimulating factor 1 receptor antibody, in combination with pembrolizumab in adults with advanced solid tumors. Immunother. Cancer.

[165] Wang X., Li S., Yan S., Shan Y., Wang X., Jingbo Z., Wang Y., Shan F., Griffin N., Sun X. (2022). Methionine enkephalin inhibits colorectal cancer by remodeling the immune status of the tumor microenvironment. Int. Immunopharmacol..

[166] Haag G.M., Springfeld C., Grün B., Apostolidis L., Zschäbitz S., Dietrich M., Berger A.K., Weber T.F., Zoernig I., Schaaf M. (2022). Pembrolizumab and maraviroc in refractory mismatch repair proficient/microsatellite-stable metastatic colorectal cancer—The PICCASSO phase I trial. Eur. J. Cancer.

[167] Han C., Deng Y., Xu W., Liu Z., Wang T., Wang S., Liu J., Liu X. (2022). The Roles of Tumor-Associated Macrophages in Prostate Cancer. J. Oncol..

[168] Armstrong A.J., Szmulewitz R.Z., Petrylak D.P., Holzbeierlein J., Villers A., Azad A., Alcaraz A., Alekseev B., Iguchi T., Shore N.D. (2019). Arches: A randomized, phase III study of androgen deprivation therapy with enzalutamide or placebo in men with metastatic hormone-sensitive prostate cancer. J. Clin. Oncol..

[169] Hoyle A.P., Ali A., James N.D., Cook A., Parker C.C., de Bono J.S., Attard G., Chowdhury S., Cross W.R., Dearnaley D.P. (2019). Abiraterone in ‘High-’ and ‘Low-risk’ Metastatic Hormone-sensitive Prostate Cancer (Figure presented.). Eur. Urol..

[170] Martori C., Sanchez-Moral L., Paul T., Pardo J.C., Font A., Ruiz de Porras V., Sarrias M.R. (2022). Macrophages as a Therapeutic Target in Metastatic Prostate Cancer: A Way to Overcome Immunotherapy Resistance?. Cancers.

[171] Davies A., Conteduca V., Zoubeidi A., Beltran H. (2019). Biological Evolution of Castration-resistant Prostate Cancer. Eur. Urol. Focus.

[172] Erlandsson A., Carlsson J., Lundholm M., Fält A., Andersson S.O., Andrén O., Davidsson S. (2019). M2 macrophages and regulatory T cells in lethal prostate cancer. Prostate.

[173] Wang C., Peng G., Huang H., Liu F., Kong D.P., Dong K.Q., Dai L.H., Zhou Z., Wang K.J., Yang J. (2018). Blocking the feedback loop between neuroendocrine differentiation and macrophages improves the therapeutic effects of enzalutamide (MDV3100) on prostate cancer. Clin. Cancer Res..

[174] Wang D., Cheng C., Chen X., Wang J., Liu K., Jing N., Xu P., Xi X., Sun Y., Ji Z. (2023). IL-1β Is an Androgen-Responsive Target in Macrophages for Immunotherapy of Prostate Cancer. Adv. Sci..

[175] Izumi K., Fang L.Y., Mizokami A., Namiki M., Li L., Lin W.J., Chang C. (2013). Targeting the androgen receptor with siRNA promotes prostate cancer metastasis through enhanced macrophage recruitment via CCL2/CCR2-induced STAT3 activation. EMBO Mol. Med..

[176] Keklikoglou I., De Palma M. (2014). Cancer: Metastasis risk after anti-macrophage therapy. Nature.

[177] Vindrieux D., Escobar P., Lazennec G. (2009). Emerging roles of chemokines in prostate cancer. Endocr. Relat. Cancer.

[178] Beyzaei Z., Shojazadeh A., Geramizadeh B. (2022). The role of regulatory T cells in liver transplantation. Transpl. Immunol..

[179] Mougel A., Adriaenssens E., Guyot B., Tian L., Gobert S., Chassat T., Persoons P., Hannebique D., Bauderlique-Le Roy H., Vicogne J. (2022). Macrophage-Colony-Stimulating Factor Receptor Enhances Prostate Cancer Cell Growth and Aggressiveness In Vitro and In Vivo and Increases Osteopontin Expression. Int. J. Mol. Sci..

[180] Luo Y., Chen Y., Jin H., Hou B., Li H., Li X., Liu L., Zhou Y., Li Y., Song Y.S. (2023). The suppression of cervical cancer ferroptosis by macrophages: The attenuation of ALOX15 in cancer cells by macrophages-derived exosomes. Acta Pharm. Sin. B.

[181] O’Donnell J.S., Teng M.W.L., Smyth M.J. (2019). Cancer immunoediting and resistance to T cell-based immunotherapy. Nat. Rev. Clin. Oncol..

[182] Shamseddine A.A., Burman B., Lee N.Y., Zamarin D., Riaz N. (2021). Tumor immunity and immunotherapy for HPV-related cancers. Cancer Discov..

[183] Kawachi A., Yoshida H., Kitano S., Ino Y., Kato T., Hiraoka N. (2018). Tumor-associated CD204+ M2 macrophages are unfavorable prognostic indicators in uterine cervical adenocarcinoma. Cancer Sci..

[184] Chen X.J., Wei W.F., Wang Z.C., Wang N., Guo C.H., Zhou C.F., Liang L.J., Wu S., Liang L., Wang W. (2021). A novel lymphatic pattern promotes metastasis of cervical cancer in a hypoxic tumour-associated macrophage-dependent manner. Angiogenesis.

[185] Chen X.J., Deng Y.R., Wang Z.C., Wei W.F., Zhou C.F., Zhang Y.M., Yan R.M., Liang L.J., Zhong M., Liang L. (2019). Hypoxia-induced ZEB1 promotes cervical cancer progression via CCL8-dependent tumour-associated macrophage recruitment. Cell Death Dis..

[186] Yang B., Chen J., Teng Y. (2021). CDK12 Promotes Cervical Cancer Progression through Enhancing Macrophage Infiltration. J. Immunol. Res..

[187] Jiang X., Stockwell B.R., Conrad M. (2021). Ferroptosis: Mechanisms, biology and role in disease. Nat. Rev. Mol. Cell Biol..

[188] Ferrandina G., Lauriola L., Distefano M.G., Zannoni G.F., Gessi M., Legge F., Maggiano N., Mancuso S., Capelli A., Scambia G. (2002). Increased cyclooxygenase-2 expression is associated with chemotherapy resistance and poor survival in cervical cancer patients. J. Clin. Oncol..

[189] Wu Y., Mao F., Zuo X., Moussalli M.J., Elias E., Xu W., Shureiqi I. (2014). 15-LOX-1 suppression of hypoxia-induced metastatic phenotype and HIF-1α expression in human colon cancer cells. Cancer Med..

[190] Mohiuddin I.S., Wei S.J., Kang M.H. (2020). Role of OCT4 in cancer stem-like cells and chemotherapy resistance. Biochim. Biophys. Acta BBA Mol. Basis Dis..

[191] Shu S., Li Z., Liu L., Ying X., Zhang Y., Wang T., Zhou X., Jiang P., Lv W. (2022). HPV16 E6-Activated OCT4 Promotes Cervical Cancer Progression by Suppressing p53 Expression via Co-Repressor NCOR1. Front. Oncol..

[192] Lu C.S., Shiau A.L., Su B.H. (2020). Oct4 promotes M2 macrophage polarization through upregulation of macrophage colony-stimulating factor in lung cancer. J. Hematol. Oncol..

[193] Bian Z., Wu X., Chen Q., Gao Q., Xue X., Wang Y. (2024). Oct4 activates IL-17A to orchestrate M2 macrophage polarization and cervical cancer metastasis. Cancer Immunol. Immunother..

[194] Thrift A.P., Wenker T.N., El-Serag H.B. (2023). Global burden of gastric cancer: Epidemiological trends, risk factors, screening and prevention. Nat. Rev. Clin. Oncol..

[195] Thrift A.P., El-Serag H.B. (2020). Burden of Gastric Cancer. Clin. Gastroenterol. Hepatol..

[196] Tang C., Lei X., Xiong L., Hu Z., Tang B. (2021). HMGA1B/2 transcriptionally activated-POU1F1 facilitates gastric carcinoma metastasis via CXCL12/CXCR4 axis-mediated macrophage polarization. Cell Death Dis..

[197] Xu J., Yu Y., He X., Niu N., Li X., Zhang R., Hu J., Ma J., Yu X., Sun Y. (2019). Tumor-associated macrophages induce invasion and poor prognosis in human gastric cancer in a cyclooxygenase-2/MMP9-dependent manner. Am. J. Transl. Res..

[198] Miao L., Qi J., Zhao Q., Wu Q.N., Wei D.L., Wei X.L., Liu J., Chen J., Zeng Z.L., Ju H.Q. (2020). Targeting the STING pathway in tumor-associated macrophages regulates innate immune sensing of gastric cancer cells. Theranostics.

[199] Park J.Y., Sung J.Y., Lee J., Park Y.K., Kim Y.W., Kim G.Y., Won K.Y., Lim S.J. (2016). Polarized CD163+ tumor-associated macrophages are associated with increased angiogenesis and CXCL12 expression in gastric cancer. Clin. Res. Hepatol. Gastroenterol..

[200] Fu L.Q., Du W.L., Cai M.H., Yao J.Y., Zhao Y.Y., Mou X.Z. (2020). The roles of tumor-associated macrophages in tumor angiogenesis and metastasis. Cell. Immunol..

[201] Guo J., Li Z., Ma Q., Li M., Zhao Y., Li B., Tao Y., Xu Y., Huang Y. (2022). Dextran Sulfate Inhibits Angiogenesis and Invasion of Gastric Cancer by Interfering with M2-type Macrophages Polarization. Curr. Cancer Drug Targets.

[202] Su P., Jiang L., Zhang Y., Yu T., Kang W., Liu Y., Yu J. (2022). Crosstalk between tumor-associated macrophages and tumor cells promotes chemoresistance via CXCL5/PI3K/AKT/mTOR pathway in gastric cancer. Cancer Cell Int..

[203] He Z., Chen D., Wu J., Sui C., Deng X., Zhang P., Chen Z., Liu D., Yu J., Shi J. (2021). Yes associated protein 1 promotes resistance to 5-fluorouracil in gastric cancer by regulating GLUT3-dependent glycometabolism reprogramming of tumor-associated macrophages. Arch. Biochem. Biophys..

[204] Gao H., Ma J., Cheng Y., Zheng P. (2020). Exosomal transfer of macrophage-derived miR-223 confers doxorubicin resistance in gastric cancer. OncoTargets Ther..

[205] Oshima H., Popivanova B.K., Oguma K., Kong D., Ishikawa T.O., Oshima M. (2011). Activation of epidermal growth factor receptor signaling by the prostaglandin E2 receptor EP4 pathway during gastric tumorigenesis. Cancer Sci..

[206] Kzhyshkowska J. (2010). Multifunctional receptor stabilin-1 in homeostasis and disease. Sci. World J..

[207] Barsouk A., Aluru J.S., Rawla P., Saginala K., Barsouk A. (2023). Epidemiology, Risk Factors, and Prevention of Head and Neck Squamous Cell Carcinoma. Med Sci..

[208] He K.F., Zhang L., Huang C.F., Ma S.R., Wang Y.F., Wang W.M., Zhao Z.L., Liu B., Zhao Y.F., Zhang W.F. (2014). CD163+ tumor-associated macrophages correlated with poor prognosis and cancer stem cells in oral squamous cell carcinoma. BioMed Res. Int..

[209] Bisheshar S.K., van der Kamp M.F., de Ruiter E.J., Ruiter L.N., van der Vegt B., Breimer G.E., Willems S.M. (2022). The prognostic role of tumor associated macrophages in squamous cell carcinoma of the head and neck: A systematic review and meta-analysis. Oral Oncol..

[210] Kansal V., Burnham A.J., Kinney B.L.C., Saba N.F., Paulos C., Lesinski G.B., Buchwald Z.S., Schmitt N.C. (2023). Statin drugs enhance responses to immune checkpoint blockade in head and neck cancer models. J. Immunother. Cancer.

[211] Zou S., Tong Q., Liu B., Huang W., Tian Y., Fu X. (2020). Targeting stat3 in cancer immunotherapy. Mol. Cancer.

[212] Moreira D., Sampath S., Won H., White S.V., Su Y.L., Alcantara M., Wang C., Lee P., Maghami E., Massarelli E. (2021). Myeloid cell-targeted STAT3 inhibition sensitizes head and neck cancers to radiotherapy and T cell-mediated immunity. J. Clin. Investig..

[213] Dan H., Liu S., Liu J., Liu D., Yin F., Wei Z., Wang J., Zhou Y., Jiang L., Ji N. (2020). RACK1 promotes cancer progression by increasing the M2/M1 macrophage ratio via the NF-κB pathway in oral squamous cell carcinoma. Mol. Oncol..

[214] Yu Z., Jiang X., Qin L., Deng H., Wang J., Ren W., Li H., Zhao L., Liu H., Yan H. (2021). A novel UBE2T inhibitor suppresses Wnt/β-catenin signaling hyperactivation and gastric cancer progression by blocking RACK1 ubiquitination. Oncogene.

[215] Jumaniyazova E., Lokhonina A., Dzhalilova D., Kosyreva A., Fatkhudinov T. (2023). Immune Cells in the Tumor Microenvironment of Soft Tissue Sarcomas. Cancers.

[216] Dancsok A.R., Gao D., Lee A.F., Steigen S.E., Blay J.Y., Thomas D.M., Maki R.G., Nielsen T.O., Demicco E.G. (2020). Tumor-associated macrophages and macrophage-related immune checkpoint expression in sarcomas. Oncoimmunology.

[217] Fan J., Qin X., He R., Ma J., Wei Q. (2021). Gene Expression Profiles for an Immunoscore Model in Bone and Soft Tissue Sarcoma. Aging.

[218] Tap W. (2020). ENLIVEN study: Pexidartinib for tenosynovial giant cell tumor (TGCT). Futur. Oncol..

[219] Giustini N., Bernthal N.M., Bukata S.V., Singh A.S. (2018). Tenosynovial giant cell tumor: Case report of a patient effectively treated with pexidartinib (PLX3397) and review of the literature. Clin. Sarcoma Res..

[220] Lamb Y.N. (2019). Pexidartinib: First Approval. Drugs.

[221] Tap W.D., Wainberg Z.A., Anthony S.P., Ibrahim P.N., Zhang C., Healey J.H., Chmielowski B., Staddon A.P., Cohn A.L., Shapiro G.I. (2015). Structure-Guided Blockade of CSF1R Kinase in Tenosynovial Giant-Cell Tumor. N. Engl. J. Med..

[222] Tang X., Mo C., Wang Y., Wei D., Xiao H. (2013). Anti-tumour strategies aiming to target tumour-associated macrophages. Immunology.

[223] Feng Y., Mu R., Wang Z., Xing P., Zhang J., Dong L., Wang C. (2019). A toll-like receptor agonist mimicking microbial signal to generate tumor-suppressive macrophages. Nat. Commun..

[224] Goff P.H., Riolobos L., LaFleur B.J., Spraker M.B., Seo Y.D., Smythe K.S., Campbell J.S., Pierce R.H., Zhang Y., He Q. (2022). Neoadjuvant Therapy Induces a Potent Immune Response to Sarcoma, Dominated by Myeloid and B Cells. Clin. Cancer Res..

